# Cerebrospinal Fluid and Brain Proteoforms of the Granin Neuropeptide Family in Alzheimer’s Disease

**DOI:** 10.1021/jasms.2c00341

**Published:** 2023-03-13

**Authors:** James P. Quinn, Elizabeth C. Ethier, Angelo Novielli, Aygul Malone, Christopher E. Ramirez, Lauren Salloum, Bianca A. Trombetta, Pia Kivisäkk, Michael Bremang, Stefan Selzer, Marjorie Fournier, Sudeshna Das, Yaoyi Xing, Steven E. Arnold, Becky C. Carlyle

**Affiliations:** † Massachusetts General Hospital Department of Neurology, Harvard Medical School, Boston, Massachusetts 02129, United States; ‡ Advanced Proteomics Facility, Department of Biochemistry, University of Oxford, Oxford, Oxfordshire OX1 3QU, United Kingdom; § Proteome Sciences LLC, Frankfurt am Main, Hessen 60438, Germany; ∥ Department of Physiology, Anatomy & Genetics, University of Oxford, Oxford, Oxfordshire OX1 3QU, United Kingdom; ⊥ Kavli Institute for Nanoscience Discovery, University of Oxford, Oxford OX1 3QU, United Kingdom

## Abstract

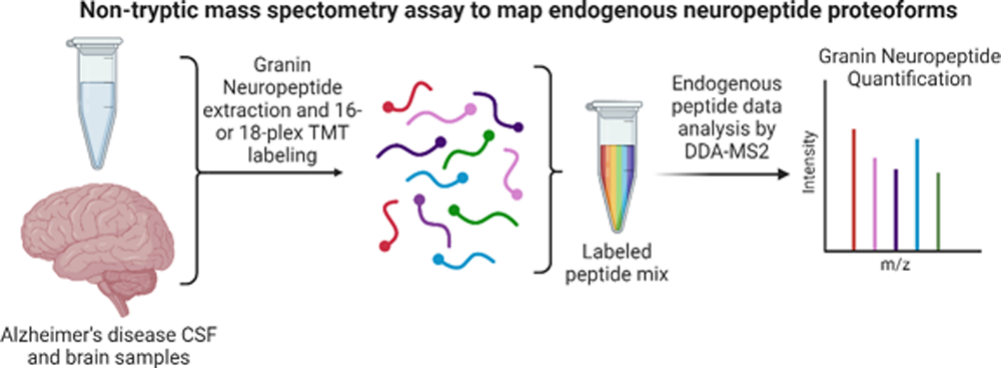

The granin neuropeptide family is composed of acidic secretory signaling molecules that act throughout the nervous system to help modulate synaptic signaling and neural activity. Granin neuropeptides have been shown to be dysregulated in different forms of dementia, including Alzheimer’s disease (AD). Recent studies have suggested that the granin neuropeptides and their protease-cleaved bioactive peptides (proteoforms) may act as both powerful drivers of gene expression and as a biomarker of synaptic health in AD. The complexity of granin proteoforms in human cerebrospinal fluid (CSF) and brain tissue has not been directly addressed. We developed a reliable nontryptic mass spectrometry assay to comprehensively map and quantify endogenous neuropeptide proteoforms in the brain and CSF of individuals diagnosed with mild cognitive impairment and dementia due to AD compared to healthy controls, individuals with preserved cognition despite AD pathology (“Resilient”), and those with impaired cognition but no AD or other discernible pathology (“Frail”). We drew associations between neuropeptide proteoforms, cognitive status, and AD pathology values. Decreased levels of VGF proteoforms were observed in CSF and brain tissue from individuals with AD compared to controls, while select proteoforms from chromogranin A showed the opposite effect. To address mechanisms of neuropeptide proteoform regulation, we showed that the proteases Calpain-1 and Cathepsin S can cleave chromogranin A, secretogranin-1, and VGF into proteoforms found in both the brain and CSF. We were unable to demonstrate differences in protease abundance in protein extracts from matched brains, suggesting that regulation may occur at the level of transcription.

## Introduction

In numerous unbiased proteomic studies, VGF (nonacronymic) has been strongly linked to neurodegenerative diseases, including Alzheimer’s disease (AD), as both a potential therapeutic target and biomarker of cognitive status and disease pathogenesis.^1−4^ Chromogranin A (CHGA) has also been strongly linked to AD, with its immunoreactivity to amyloid plaque pathology^5^ and potential as a biomarker that differentiates AD from controls.^6^ VGF and CHGA are members of the granin family of neuropeptides, acidic secretory proteins,^7^ that also include the Secretogranins-1, -2, -3, and -5 (SCG-1, -2, -3, and -5). These range from 212 to 677 amino acids in size and have a signal peptide on the N-terminus ranging from 18 to 27 amino acids,^7^ which is removed to help mediate neuropeptide secretion via the regulated secretory pathway.^8−10^ Neuropeptides have been shown to be trafficked from the Golgi apparatus through large dense core vesicles, where they are processed by proteases, including cathepsins, metalloproteases, and prohormone convertases,^11^ from their inactive precursor proteins into their functional active neuropeptide proteoforms.^1^ Neuropeptides exist throughout the nervous system to help modulate synaptic signaling, neural activity, and the activity of other organs throughout the body.^10,12,13^ VGF has been shown to play a critical role in regulating synaptogenesis and neurogenesis which may regulate energy balance, learning, and memory.^1,14−16^

Established biofluid biomarkers for AD can accurately quantity brain levels of Amyloid-β Aβ_40_, Aβ_42_, phosphorylated Tau 181 (pTau 181), and total Tau, but these biomarkers may be less sensitive to cognitive changes.^17−20^ Up to one-third of Amyloid, Tau, and Neurodegeneration (ATN) positive individuals may exhibit cognitive resilience, experiencing minimal decline in cognitive performance despite a significant burden of AD pathology.^21−24^ Post-mortem synaptic density is one of the strongest correlates of cognitive performance,^25−27^ and given their established functions, granin peptides may represent informative biomarkers of both synaptic health and cognitive status.

In this study we first aimed to comprehensively map endogenous neuropeptide proteoforms in the brain and CSF of patients with divergent AD pathology and cognitive status. We identified novel endogenous peptides from all target granin family members. Second, we converted these identifications into robust quantitative mass spectrometry (MS) assays utilizing tandem mass tag (TMT) labeling to associate endogenous neuropeptide proteoforms with cognitive classification in both CSF and brain. In general, VGF peptides decrease in CSF as cognitive classification worsens, but the relationship between VGF, pathology, and cognitive classification in brain is more complex. In brain tissue, but not in CSF, select peptides from CHGA were higher in AD-Dementia or Frail individuals.

To address whether granin neuropeptides were regulated at the transcriptional level, we used immunoblotting to examine full-length neuropeptide levels in brain tissue. Full-length CHGA was increased in cognitively impaired individuals with AD-pathology, while VGF was decreased in all individuals with significant AD-pathology. Finally, to investigate the potential for post-translational regulation of neuropeptides, we used in-silico and in vitro protease digestions to link specific protease enzymes to novel peptide proteoforms. Calpain-1 and Cathepsin S were established as potential protease enzymes, but no between-group abundance differences were observed in either protease. We therefore further add to the current literature which suggests that regulation of neuropeptides may be primarily at the transcript level, and not via altered protease activity.

## Methods

### Cerebrospinal Fluid Study Participants

Cerebrospinal fluid (CSF) samples were selected from the Massachusetts General Institute for Neurodegenerative Disease (MIND) and LifeSPAN biorepositories under Institutional Review Board (IRB) Protocols 2015P000221 and 2018P001989. All subjects provided written informed consent. MIND samples were obtained from diagnostic lumbar punctures from the Department of Neurology at Massachusetts General Hospital between 2014 and 2020. Samples were collected into polypropylene collection tubes and aliquoted and frozen on dry ice within 30 min of sample collection. Samples were stored at −80 °C until use. CSF Amyloid positive individuals were divided into groups by an experienced neurologist and psychiatrist (SEA) based on their cognitive status into asymptomatic (Asymp-AD, no cognitive impairment at time of lumbar puncture), mild cognitive impairment (MCI), and dementia (AD-Dementia). The cognitive scores available were not identical across all participants due to samples being obtained from a diagnostic clinic but included a combination of clinical dementia rating (CDR), Montreal Cognitive Assessment (MOCA), and Mini Mental State Examination (MMSE). Euroimmun (Lübeck, Germany) assays for CSF Aβ_40_, Aβ_42_, pTau 181, and total Tau were performed according to the Manufacturer’s Instructions on a Tecan Freedom Evo liquid handler (Zürich, Switzerland). Participants were classified as Amyloid positive according to an in-house established thresholds of Aβ_40_/Aβ_42_ ratio below 0.082 (sensitivity = 0.92, specificity = 0.91). Summary demographic information about the CSF cohort is provided in Table 1; individual level data is provided in Supplementary Table 1.

**Table 1:** Demographics of CSF Samples^a^

	AD-DEM (*N* = 47)	AD-MCI (*N* = 63)	AD-Asymp (*N* = 23)	HC (*N* = 32)	Total (*N* = 165)
**Age (years)**					
Mean (SD)	68.9 (9.67)	68.7 (8.45)	72.8 (8.60)	65.7 (7.90)	68.7 (8.89)
**Sex**					
Female	20.0 (42.6%)	29.0 (46.0%)	14.0 (60.9%)	14.0 (43.8%)	77.0 (46.7%)
**Aβ**_**42**_**/Aβ**_**40**_					
Mean (SD)	0.050 (0.013)	0.047 (0.012)	0.049 (0.015)	0.128 (0.038)	0.064 (0.037)
**pTau 181 (pg/mL)**					
Mean (SD)	123 (48.2)	107 (35.1)	64.1 (29.7)	28.0 (6.75)	91.4 (50.3)
**Total Tau (pg/mL)**					
Mean (SD)	568 (263)	504 (154)	336 (146)	191 (46.6)	438 (227)
**MoCA**					
Mean (SD, *n*)	14.3 (6, 15)	21.2 (4.7, 29)	26 (NA, 1)	27.7 (2.1, 19)	21.6 (6.5, 64)
**MMSE**					
Mean (SD, *n*)	21.6 (3.8, 8)	25.1 (2.9,11)	29 (NA, 1)	30 (NA, 1)	24.2 (3.9, 21)

a Because samples are selected from cohorts with varying cognitive testing measures, there is no uniform cognitive score across the cohort. The MoCA and MMSE scores that are available are presented to show the average cognitive status of each group.

### Brain Tissue Study Participants

Post-mortem tissue from the parietal cortex (angular gyrus, Brodmann Area 39) was obtained from the Rush Alzheimer’s Disease Center. This region was selected as it is affected by encroaching Aβ and Tau pathology at intermediate stages of the Braak staging spectrum for AD.^28,29^ Tissue came from both the Rush Religious Orders Study (ROS) and Memory and Aging (MAP) projects,^30^ which are longitudinal cohort studies that perform comprehensive yearly cognitive testing,^31,32^ clinical evaluations, and informed consent given for brain donation for research at the time of death. Scores from 19 cognitive tests were converted to Z-scores based on the baseline mean and standard deviation across the entire ROSMAP cohort (>1700 individuals) and combined to create a composite global cognition score. Brain autopsies were conducted according to standard protocols, including the preparation of diagnostic blocks from multiple brain regions for neuropathological classification according to NIA-Reagan, Braak, and CERAD staging. Written informed consent was obtained from all participants, and tissue was obtained and analyzed under an Exempt Secondary Use IRB protocol approved by the Massachusetts General Hospital IRB (2016P001074). The 102 participants were divided into four categorical groups of 25 or 26 samples each as previously published^33^ on the basis of their Braak neuropathology stage^34^ (Braak score of ≤4, low pathology, Braak score of >4, high pathology) and consensus clinical cognitive diagnosis from longitudinal cohort clinicians (impaired/unimpaired). Samples were selected in quartets, allowing for pairing of individuals with the same levels of pathology but divergent cognitive status. Resilient individuals have high AD pathology but intact cognitive performance. Frail individuals (low pathology, cognitive impairment) exhibited no greater TDP43, Lewy body or vascular pathology than the other three groups. Summary demographic information for the brain tissue cohort is provided in Table 2; individual level demographic information in Supplementary Table 2.

**Table 2:** Sample Demographics for Brain Tissue Study

	Control (*N* = 26)	AD-DEM (*N* = 25)	Frail (*N* = 26)	AD-Resilient (*N* = 25)	Total (*N* = 102)
**Age (years)**					
Mean (SD)	90.6 (5.29)	90.4 (5.32)	88.0 (6.56)	91.0 (5.19)	90.0 (5.67)
**Sex**					
Female	15.0 (57.7%)	14.0 (56.0%)	13.0 (50.0%)	14.0 (56.0%)	56.0 (54.9%)
**Education (years)**					
Mean (SD)	17.1 (3.69)	16.2 (3.22)	16.8 (3.46)	16.4 (3.87)	16.6 (3.53)
**Global pathology** (z-score)					
Mean (SD)	0.275 (0.38)	1.61 (0.61)	0.219 (0.3)	1.28 (0.53)	0.834 (0.77)
**Global cognition** (z-score)					
Mean (SD)	0.30 (0.33)	–2.19 (1.03)	–1.46 (0.74)	–0.097 (0.34)	–0.86 (1.21)

### Cerebrospinal Fluid Sample Preparation for Mass Spectrometry

Upon receipt by Proteome Sciences (Surrey, UK), CSF samples were visually inspected for tube integrity and absence of thawing. Prior to analysis by MS, the protein concentration of each sample was determined using a modified Bradford assay (Thermo Fisher Scientific, Waltham, MA), and 10 μg of each sample was visualized on Coomassie (Imperial Stain, Thermo Fisher Scientific)-stained sodium dodecyl sulfate–polyacrylamide gel electrophoresis (SDS–PAGE) 4–20% gradient gels (Criterion, Bio-Rad, Hercules, CA) to assess overall proteome integrity. No samples showed clear signs of widespread degradation. For each single sample assessment (volumes were scaled for pooled experiments), 250 μL of CSF was diluted to a final concentration of 2.4 M guanidium chloride and 100 mM triethylammonium bicarbonate (TEAB). Cysteines were reduced by treatment with 1 mM tris(2-carboxyethyl)phosphine hydrochloride (TCEP) for 1 h at 55 °C followed by alkylation with 7.5 mM iodoacetamide for 1 h at room temperature. Samples were tandem mass tag (TMT) labeled using 25 mM TMTpro reagents (Thermo Fisher Scientific) for 1 h at room temperature. After treatment with hydroxylamine at 0.75% for 30 min at room temperature, samples were combined into TMTpro 16 plexes, then were processed through 30 kDa ultrafiltration cartridges (Amicon, Merck KGaA, Darmstadt, Germany). The flow-through was subjected to further peptide purification by solid phase extraction using OASIS HLB (Waters, Milford, MA) and SP Sepharose Fast Flow (Cytiva, Marlborough, MA) resin. Specifically, samples were diluted with 0.1% trifluoroacetic acid (TFA) to reduce the acetonitrile (ACN) content to <5% and desalted using HLB Oasis cartridges with the aid of a vacuum manifold. Bound peptides were washed with 5% ACN, 0.1% TFA and eluted with 50% ACN, 0.1% TFA. The eluates were loaded onto self-made cartridges (CHROMABOND empty columns 15 mL, Macherey-Nagel, filled with 650 μL SP Sepharose Fast Flow, Sigma) and, after washing with 25% ACN, 0.1% TFA, the peptides were eluted with 25% ACN, 400 mM ammonium acetate. The eluate was dried, and samples stored at −80 °C prior to analysis.

### CSF Mass Spectrometry Data Acquisition

#### Experiment 1: Deep Mapping of Nontryptic Neuropeptide Proteoforms

To produce a deep map of neuropeptide proteoforms present in CSF, a mixed reference pool of 10 CSF samples (*n* = 5 AD, *n* = 5 Control) was produced by mixing equal volumes of each sample. This reference pool was analyzed by single-shot acquisition and included on a TMTPro 11plex in addition to each individual sample. The TMTPro 11plex was separated into 15 fractions by basic reversed-phase chromatography (NUCLEOSIL 120-4 C18 column) on a high-performance liquid chromatography (HPLC) system (Waters Alliance 2695), and the 15 fractions were combined into 6 analytical fractions. All samples were analyzed by liquid chromatography–MS/MS (LC–MS/MS) on an Orbitrap Fusion Tribrid mass spectrometer coupled to an EASY-nanoLC 100 system (Thermo Fisher Scientific). Peptides were loaded onto a nanoViper C18 Acclaim PepMap 100 precolumn (Thermo Fisher Scientific) and resolved using an increasing gradient of ACN in 0.1% formic acid (FA) through a 50 cm PepMap RSLC analytical column (Thermo Fisher Scientific) at a flow rate of 200 nL/min. An increasing ACN gradient was applied over 160 min (from 5% to 30/40% depending on sample), rising to 90% ACN at a flow rate of 300 nL/min for an additional 20 min. Mass spectra were acquired throughout the entire chromatographic run, using top speed higher collision induced dissociation (HCD) Fourier-transform mass spectrometry (FTMS) MS2 scans at 60,000 resolving power at 400 mass over charge (*m*/*z*), following each FTMS scan (120,000 resolving power at 400 *m*/*z*).

#### Experiment 2: Linearity, Robustness, and Limit of Detection

Pooled reference CSF was used to assess quantitative performance of the assay. To determine the linearity and limit of detection of neuropeptide proteoforms, two seven-point dilution series (neat, 1/2, 1/4, 1/8, 1/16, 1/32, 1/64 of pooled CSF were prepared in PBS buffer containing 0.05% bovine albumin). To quantify assay multiday reproducibility, 16 aliquots of 0.2 mL of pooled reference CSF were each prepared on three separate days. All samples were analyzed using the EASY-nLC 1000 system coupled to an Orbitrap Fusion Tribrid Mass Spectrometer (both Thermo Fisher Scientific) applying a semitargeted data-dependent acquisition (DDA) method with an inclusion list for the neuropeptide proteoform targets of interest. Resuspended peptides were loaded onto a nanoViper C18 Acclaim PepMap 100 trap column (PN 164946, Thermo Fisher Scientific) and resolved using an increasing gradient of ACN in 0.1% FA through a 50 cm 75 μm ID EasySpray analytical column (PN ES803A, Thermo Fisher Scientific) at a flow rate of 200 nL/min during the first 160 min and 300 nL/min over the last 20 min. The LC gradient started at 5% ACN and increased linearly up to 40% ACN over the first 160 min, then 90% ACN was held constant over the last 20 min. Peptide mass spectra were acquired according to the *m*/*z* and charge state of 265 precursors (TMTpro labeled) detailed in a target mass list. Whenever a targeted *m*/*z* feature was detected in an MS1 full scan and passed the intensity threshold of 5e3, an MS2 dependent scan was triggered. Peptide precursors were isolated in a 1.2 Th (Thomson) wide isolation window and fragmented by higher collision-induced dissociation (HCD) at a normalized collision energy of 38%. Full scans were acquired at 120 K resolution, while fragment scans were acquired at 30K resolution. The automatic gain control (AGC) target for dependent scans was set to 1e5 with a maximum injection time of 54 ms. An inclusion list was created from the peptide species identified in the mapping experiment.

#### Experiment 3: Quantification of Neuropeptide Proteoforms in a 165 Participant Cohort

250 μL from each of the 165 individual CSF samples were processed in five batches and randomized to 16 TMTpro 16 plexes (Supplementary Table 3). Each 16 plex contained 2 reference pooled CSF samples. Data acquisition was performed using a semitargeted data-dependent MS2 acquisition method with an inclusion list generated from all previously observed neuropeptide proteoforms. Samples were analyzed using the EASY-nLC 1000 system coupled to an Orbitrap Fusion Tribrid Mass Spectrometer as per experiment 2 above.

### CSF Peptide Data Processing

Across all experiments, raw files were submitted to Proteome Discoverer (PD) (Ver 2.1, Thermo Fisher Scientific) using the Spectrum Files node. The reporter ions quantifier node was set up to measure the raw intensity values for TMT plex monoisotopic ions. The Spectrum Selector was set to its default values. All spectra passing these filters were exported in MGF-format before and after removing TMT tag fragments for further processing. Spectral clustering was performed using MS-Cluster (Ver 2). The following parameters were used for clustering: sqs = 0.1, mixture probability 0.05 and fragment tolerance 0.02. Identified clusters were then intersected with quanSpectra output from the original PD processing, to generate a suitable file format for downstream bioinformatics processing.

#### Experiment 1: Deep Mapping of Nontryptic Neuropeptide Proteoforms

Clusters related to quantified peptides were searched on PD 2.1 using SEQUEST-HT for database searching. The Spectrum Selector was set to its default values, while the SEQUEST-HT node was suitably set up to search data against a human FASTA UniProtKB/Swiss-Prot database (Ver Feb 2019). The SEQUEST-HT search engine was programmed to search for peptides with protease specificity set to “no enzyme” and with carbamidomethyl (Cysteine) and TMT (N-terminal; Lysine) set as static modifications. Precursor mass tolerance was set to 20 parts per million (ppm) and fragment (b and y ions) mass tolerance to 0.02 Da. Grouped protein results were exported, filtered at 1% false discovery rate (FDR) on peptide spectral match (PSM) and 1 x Rank 1 peptide per protein, based on results from the Percolator PD node. In parallel, PEAKS Studio (Ver 7.5, BSI, Ontario, Canada) was used to add additional confidence in identification. The search settings were: precursor Δm tolerance = 10 ppm, fragment Δm tolerance = 20 milli mass units, carbamidomethyl (Cysteine) and TMT (N-terminal; Lysine) set as static modifications, searching the human FASTA UniProtKB/Swiss-Prot database (Ver Feb 2019). Automatic de novo sequencing was performed by the autode novo function in PEAKS Studio and the DB-Search function enabled assignment of peptide sequences and protein identifiers. Across all proteins, 6609 spectral clusters, (5,466 in all samples), were identified in the 180 min gradient single-shot unfractionated analysis, with 25,786 clusters (17,134 in all samples) detected in the fractionated analysis.

#### Experiment 2: Linearity, Robustness, and Limit of Detection

Spectra were searched against a human UniProtKB database (Ver Jan. 13th, 2020) using the SEQUEST-HT search algorithm. The enzyme specificity was set to “no enzyme”, and the precursor and fragment tolerances were set to 10 ppm and 0.05 Da, respectively. Oxidation of methionine was set as a variable modification, and carbamidomethyl (Cysteine) and TMT (N-terminal; Lysine) were set as static modifications. Precursor tolerance was set to 10 ppm, and the fragment ion tolerance was set to 0.05 Da. Search engine results were filtered to 1% FDR on PSM level by the Percolator node within PD.

#### Experiment 3: Quantification of Neuropeptide Proteoforms in a 165 Participant Cohort

Spectral searching was performed as per Experiment 2.

### CSF Neuropeptide Proteoform Quantification

All data was processed initially in Proteome Sciences proprietary DIANA software. Isotope impurity correction was applied to PSM level data to address impurities due to isotopic overlap of the different reporter ion masses. Isotope correction factors were specific to the batch of TMT reagents used for labeling. PSM with isolation interference higher than 50% was removed.

#### Experiment 2: Linearity, Robustness, And Limit of Detection

Intensities of the reporter ions of each sample were median-scaled, and then ratios of reporter ion intensities were calculated for experimental samples relative to the reference sample and log2- transformed. For the linearity experiment, the highest calibration point (Neat) for each of the Dilution A and B were used for the ratio calculation. For each peptide the linear model
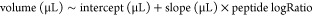
was fitted, and the coefficient of determination (*R*^2^) was extracted as a measure of dilution linearity. For the robustness experiment, the experiment median of the samples were used. Data belonging to identical peptide sequences were summarized to transform the PSM data matrix into a peptide matrix.

#### Experiment 3: Quantification of Neuropeptide Proteoforms in a 165 Participant Cohort

Normalization factors specific to each TMT channel were computed on the subset of robustly measured (S/*N* > 10; Isolation Interference <50%) unmatched peptidic spectra and used to adjust matched spectra. For each channel within a TMT plex, a channel-average value was computed as the median logRatio across all peptides. Normalization factors were then determined as adjusted channel-average values (calculated relative to the median across all channel-average values) and used to normalize reporter ion intensities for the neuropeptide proteoforms of interest.

### Brain Tissue Sample Preparation for Mass Spectrometry

Human post-mortem brain tissue was lysed by sonication in 4 M guanidine hydrochloride in 100 mM TEAB. The protein concentration of each sample was determined using a modified Bradford assay, and 10 μg of each sample was visualized on Coomassie stained SDS-PAGE 4–20% gradient gels to assess overall proteome integrity. No samples showed clear signs of widespread degradation. 600 μg of each lysate was adjusted to equal concentration with 4 M Guanidine hydrochloride in 100 mM TEAB. A reference pool was produced by mixing 100 μg of each sample with the remaining 500 μg used as individual analytic samples. Samples were TMT labeled using 25 mM TMTpro reagents for 1 h at room temperature. The samples were pooled into six analytical TMTpro 18 plexes of approximately 9 mg of protein per plex (Supplementary Table 4). The pooled samples were processed through 50 kDa ultrafiltration cartridges (Amicon) to enrich for endogenous peptides. The flow-through was reduced by treatment with TCEP for 1 h at room temperature followed by alkylation with 7.5 mM iodoacetamide for 1 h at room temperature and treatment with hydroxylamine at 0.75% for 30 min at room temperature. Samples were purified by solid-phase extraction using OASIS HLB (Vac RC 30 mg, Waters). Specifically, samples were diluted with 3 volumes of 5% ACN 0.1% TFA to reduce the ACN content to <5% and desalted using HLB Oasis cartridges with the aid of a vacuum manifold. Bound peptides were washed with 5% ACN, 0.1% TFA and eluted with 50% ACN, 0.1% TFA. The eluate was split into two parts; 10% for labeling efficiency and equimolarity checks and 90% for analysis and dried in a SpeedVac. Samples for analysis were fractionated using the Pierce High pH Reversed-Phase Peptide Fractionation kit (Thermo Fisher Scientific) according to Manufacturer’s instructions. Fractions 1 and 2 and 7 and 8 were combined to produce 6 fractions total which were dried in a SpeedVac and stored at −80 °C prior to analysis.

### Immunoblot Analysis of Brain Tissue Samples

84 of the 102 ROSMAP brain tissue samples used for mass-spectrometry (*n* = 21 Control, *n* = 20 AD-Dementia, *n* = 22 Frail, and *n* = 21 AD-Resilient) were available for immunoblotting and prepared by sonicating approximately 200 mg (250 mg/mL) of tissue in RIPA lysis buffer (Thermo Fisher Scientific) plus cOmplete Mini protease and PhosSTOP phosphatase inhibitors (both Roche Diagnostics GmbH, Mannheim, Germany). Samples were centrifuged at 14,000*g* for 10 min at 4 °C, the concentration was determined by modified Bradford assay, and 20 μg samples were prepared by the addition of 5× protein loading buffer (National Diagnostics, Atlanta, GA) and heating at 95 °C for 5 min. A pooled reference sample was produced by mixing equal volumes of each sample to allow comparison between blots. Proteins were separated using SDS–PAGE Tris-Glycine 4–20% or 10–20% gradient gels (Thermo Fisher Scientific), transferred to a 0.45 μm nitrocellulose membrane (Bio-Rad), blocked for 1 h at room temperature in Intercept PBS blocking buffer (LI-COR Biosciences, Lincoln, NE), and stained with Calpain-1, Cathepsin S, Chromogranin A, Secretogranin-1, -2, -3, -5, and VGF antibodies overnight at 4 °C followed by fluorescently conjugated secondary antibodies for 1 h at room temperature (Supplementary Table 5). Blots were imaged on an ODYSSEY CLx (LI-COR Biosciences). Precision plus protein dual color standards molecular weight ladder (10–250 kDa; Bio-Rad) were used. Samples were randomized, separated into seven groups and blinded to the investigator.

### Immunoblot Analysis of Full-Length Neuropeptide and Protease Levels

Blots were analyzed using Image Studio Lite (LI-COR Biosciences, Ver 5.2). Full-length neuropeptide and protease background intensity normalized signal values were obtained and expressed relative to the pooled reference sample for each immunoblot; these values were then plotted as a ratio to the amido black stain signal intensity in each band to account for total protein. Values were plotted on Prism (GraphPad Software, San Diego, CA, Ver 9.4.1), and statistical analysis was performed by first assessing for normality of the data using the D’Agostino and Pearson test. This was followed by either the One-Way ANOVA with the Tukey’s multiple comparisons test for data with a normal distribution or the Kruskal–Wallis test with the Dunn’s multiple comparisons test for data without a normal distribution. An adjusted *p* value below 0.05 was considered as significant.

### Bioinformatics to Predict Protease Cleavage of Neuropeptides

All CSF and brain neuropeptide proteoforms were uploaded to the PROTEASIX (Ver Jan 2017) online peptide-centric prediction tool to predict potential proteases that cleave at the N- or C-terminus of the neuropeptide proteoforms from the MEROPS database. The results generated from this search were matched against protease expression levels in the brain at the protein and RNA level using information from the human protein atlas (https://www.proteinatlas.org/, Version 21).

### In Vitro Protease Cleavage Assays of Neuropeptides

The Calpain-1 digestion assay was performed for 10 min at 30 °C and was stopped by freezing at −80 °C prior to MS analysis. The reaction was performed in calpain digestion buffer in a reaction volume of 15 μL (6 mM CaCl2, 4 mM DTT, 50 mM NaCl, 50 mM Tris, pH 7.5) using 1 μg of recombinant human VGF (TP309477, Origene, Rockville, MD), CHGA (AB85486, Abcam, Cambridge, UK), SCG1 (AB93930, Abcam), and 20 ng of recombinant human Calpain-1 (AB91019, Abcam) diluted in the calpain digestion buffer.

The Cathepsin S digestion assay was performed for 1 h at 37 °C and was stopped by freezing at −80 °C prior to MS analysis. The reaction was performed in PBS in a reaction volume of 15 μL using 1 μg of recombinant human VGF, CHGA, and SCG1 and 10 ng of recombinant human Cathepsin S (219343-25UG, MilliporeSigma, Darmstadt, Germany) diluted in PBS.

All digestion assays were performed in triplicate, and for immunoblot analysis of these samples, 1 μL of the total reaction volume was diluted in 1× loading buffer to 50 μL, and 15 μL of each reaction was separated using SDS–PAGE as described above.

### Mass Spectrometry to Identify Protease Cleavage Sites within Neuropeptides

14 μL of the total 15 μL reaction volume from the recombinant protease experiments (± protease) was denatured in 8 M urea/0.1 M ammonium bicarbonate, reduced with 10 mM TCEP, and alkylated with 50 mM 2-chloroacetamide. Peptides were further digested with trypsin at a ratio of 1:40. Digested peptides were solid-phase extracted using C18 stage-tips, and the eluate was dried under vacuum. Peptide pellets were resuspended in loading buffer (5% ACN/5% FA) for separation and identification by LC–MS/MS. Peptides were separated by nano liquid chromatography (Thermo Scientific Ultimate RSLC 3000) coupled in line to a Q Exactive mass spectrometer equipped with an Easy-Spray source (Thermo Fisher Scientific). Peptides were trapped onto a C18 PepMac100 precolumn (300 μm i.d. × 5 mm, 100 Å, Thermo Fisher Scientific) using Solvent A (0.1% FA, HPLC grade water). The peptides were further separated onto an Easy-Spray RSLC C18 column (75 μm i.d., 50 cm length, Thermo Fisher Scientific) using a 15 min linear gradient (15% to 38% solvent B (0.1% FA in ACN)) at a flow rate of 200 nL/min. The raw data were acquired on the mass spectrometer in a DDA mode. Full-scan MS spectra were acquired in the Orbitrap (Scan range 350–1500 *m*/*z*, resolution 70,000; AGC target, 3e6, maximum injection time, 50 ms). The five most intense peaks were selected for HCD fragmentation at 30% of normalized collision energy. HCD spectra were acquired in the Orbitrap at resolution 17,500, AGC target 5e4, maximum injection time 120 ms with fixed mass at 180 *m*/*z*. Charge exclusion was selected for unassigned and 1+ ions. The dynamic exclusion was set to 5 s.

### LC–MS/MS Protocol to Identify Protease Cleavage Sites within Neuropeptides

Tandem mass (MS/MS) spectra were searched using Sequest HT in Proteome Discoverer (Ver 1.4) as follows: MS/MS data from samples were searched against a protein sequence database containing 286 protein entries, including human reference sequences for CHGA, SCG1, and VGF, and for 283 common contaminants. During database searching cysteines were considered to be fully carbamidomethylated (+57,0215, statically added), methionine to be fully oxidized (+15,9949, dynamically added), all N-terminal residues to be acetylated (+42,0106, dynamically added). Database searching was extended to semitryptic peptides to identify the peptides with at least one nontryptic cleavage end (semitryptic search). Two missed cleavages were permitted. Peptide mass tolerance was set at 50 ppm on the precursor and 0.6 Da on the fragment ions. Data was filtered at FDR below 1% at PSM level.

### Modeling and Statistical Analysis

Downstream analysis and figure plotting from MS data was performed in R using the tidyverse, HMisc, broom, UpSetR, table1, ggsignif, lmtest, and glmnet packages. For both CSF and brain modeling, peptides with greater than 20% missing values were excluded from the data set, and missing values were not imputed (i.e., kept as NA). For both analyses, a linear model was fit as follows for each peptide:
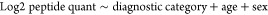
*p* values were multiple-test corrected using the Benjamini–Hochberg procedure, and an adjusted *p* value below 0.05 was considered significant. For correlation plots, missing values were replaced by 0. To select peptides for inclusion in stepwise predictive modeling of cognitive performance, an elastic net was used, which included global pathology score (a composite score encompassing both amyloid and Tau pathology), age and sex as unpenalized variables, and all peptides as penalized variables. Models were tested across a range of alpha and lambda values. Peptides from the leading model were ranked by coefficient and added stepwise to the base linear regression model
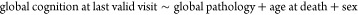
until the new model failed to produce a significant *p* value in a likelihood ratio test.

## Results

### High-Resolution Mapping of CSF Neuropeptide Proteoforms

**Figure 1:**
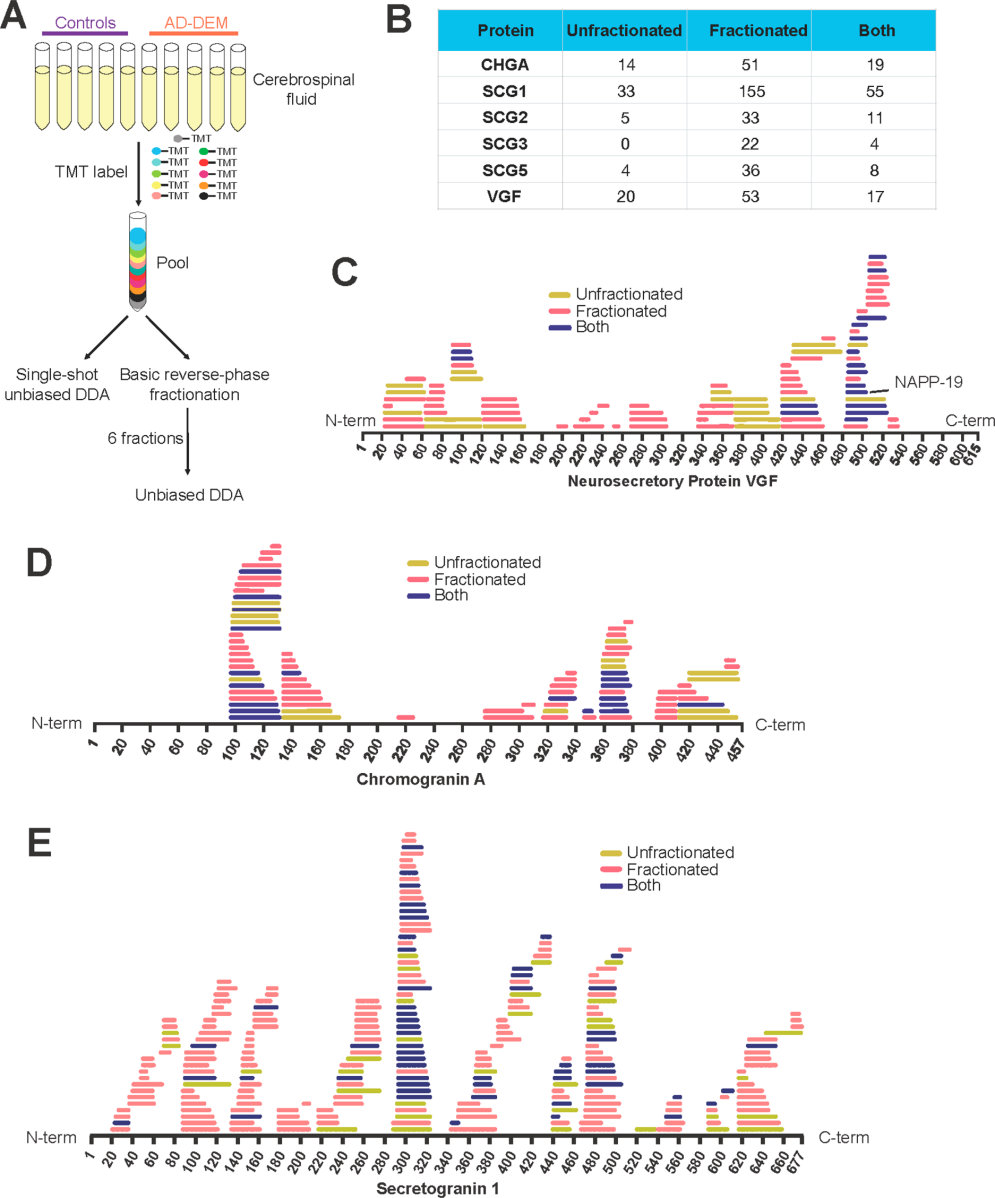
Comparison of fractionated and unfractionated CSF analysis of neuropeptide proteoforms. (A) Schematic diagram showing experimental setup for deep mapping of CSF neuropeptide proteoforms. (B) Table showing the number of target neuropeptide proteoforms identified in only unfractionated or fractionated samples or in both sample types. (C) Map of neuropeptide proteoforms detected in VGF, (D) chromogranin A, and (E) secretogranin 1. Neuropeptide proteoforms identified only in unfractionated samples are shown in yellow and identified only in fractionated samples in pink, and proteoforms identified by both analyses are shown in dark blue.

To produce a comprehensive map of endogenous neuropeptide proteoforms present in human CSF, peptides from *n* = 5 AD and *n* = 5 control participants were analyzed by data-dependent acquisition-mass spectrometry/mass spectrometry (DDA-MS2) with and without preceding basic-reversed phase fractionation (6 analytical fractions, Figure 1A). Nontryptic neuropeptide proteoforms were identified using a parallel approach that combined spectral searching in both Proteome Discoverer and PEAKS Studio. 76 proteoforms from VGF, CHGA, SCG-1, -2, -3, and -5 were identified in the unfractionated analysis, while 350 were identified in the fractionated analysis alone. 114 were identified in both unfractionated and fractionated analysis (Figure 1B). Although more proteoforms were identified in the fractionated analysis, there was a significant degree of overlap between groups (Figure 1C–E, Supplementary Figure 1A–C, and Supplementary Table 6). Of the established VGF proteoforms, we identified VGF_485–503_ (NAPP-19/NERP-4) in both fractionated and unfractionated analyses (Figure 1C) and did not find evidence for any of the VGF TLQP proteoforms (TLQP-21/TLQP-62). Many proteoforms cluster into groups with the same N-terminal amino acid start site, suggesting multiple C-terminal truncations of the same N-terminal proteoform are present. Despite the lower overall coverage seen in the unfractionated samples, most clusters are represented by at least one proteoform in the unfractionated analysis, with the significant exception of VGF proteoforms between amino acids 170–320. For this reason, unfractionated single-shot analysis was selected for quantitative experiments.

### Assessment of Peptide Linearity, Sensitivity, and Reproducibility in Quantification Assay

**Figure 2:**
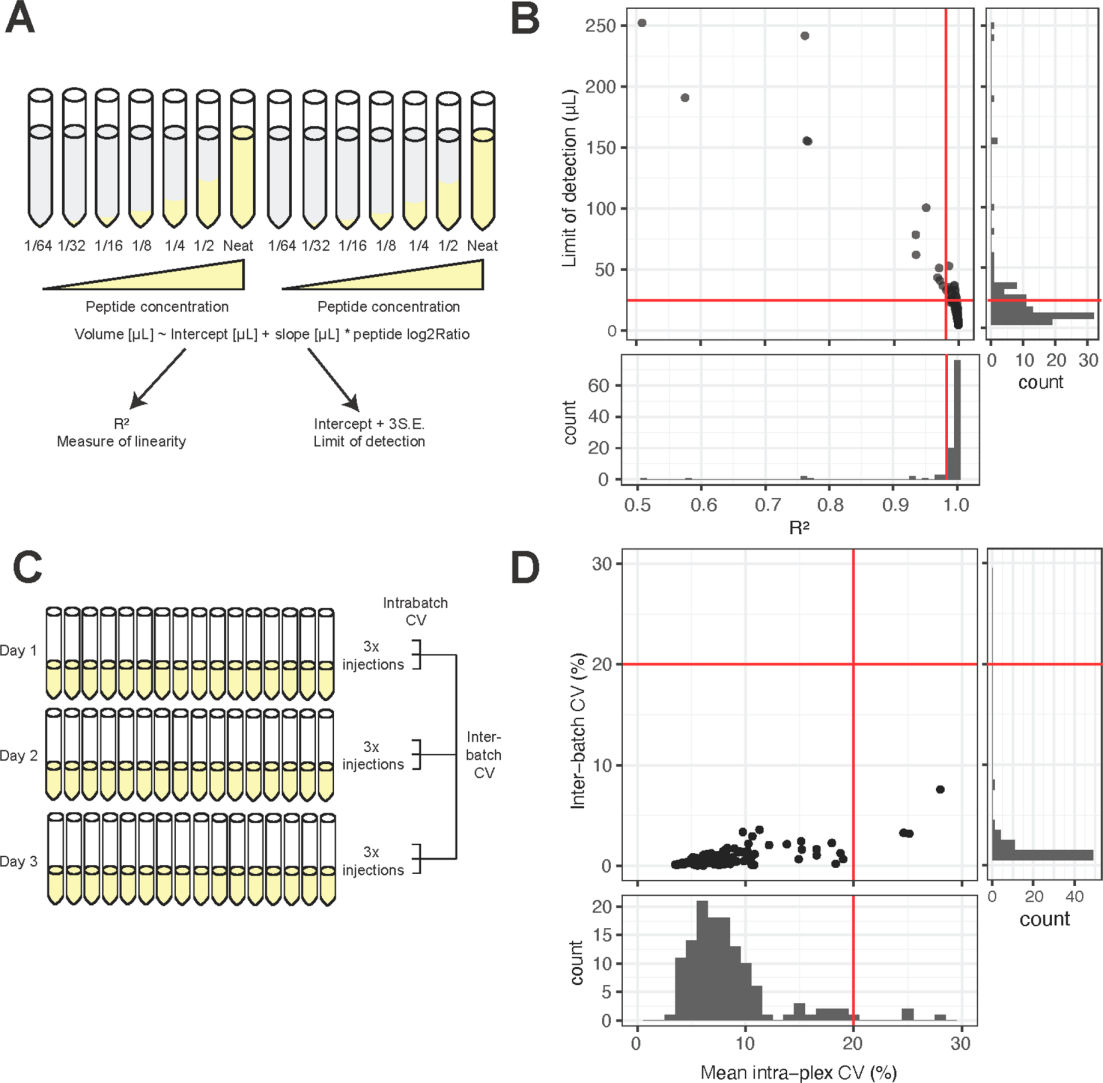
Cerebrospinal fluid neuropeptide proteoforms linearity and robustness. (A) Experimental scheme for the CSF peptide linearity experiment. (B) The majority of peptides analyzed show excellent linearity above an *R*^2^ of 0.98 and are detectable in less than 25 μL of CSF (i.e., a limit of detection below 25 μL). The scatter plot shows that neuropeptide proteoforms that require a higher input volume to be detected also demonstrate poor linearity. (C) Experimental scheme for the peptide reproducibility experiment. (D) The majority of peptides identified are robustly quantified, with Intraplex and Interbatch CVs below 20%.

To ensure accurate quantification of the maximum number of neuropeptide proteoforms, multiple rounds of MS testing and optimization were performed. To assess peptide linearity, a DDA-MS2-targeted method was used to analyze two biological replicate 7-point dilution series of pooled reference CSF (from neat to 1/64 dilution) on a single 14-plex TMTpro run (Figure 2A, Supplementary Table 7). 110 target peptides were identified in at least one dilution series. 98 peptides had an *R*^2^ greater than 0.98 across both dilution series (Figure 2B). The limit of detection was calculated^35^ as the linear model intercept plus 3 times standard error of the intercept. 100 peptides were detectable at 50 μL or below. As expected, at extreme values, peptide linearity was associated with the limit of detection (LOD), with those samples with highest LODs showing the worst linearity (Figure 2B).

To address peptide reproducibility, three TMTpro 16-plexes containing equal amounts of the same pooled reference CSF peptides were prepared on three different days. Each 16-plex was analyzed in triplicate injections using the DDA-MS2 acquisition method (Figure 2C). 128 peptides from the target proteins were identified in this experiment (Supplementary Table 8). Peptide reproducibility was excellent, with 125 peptides exhibiting an intrabatch CV below 20% (Figure 2D, Supplementary Table 9). As with poor linearity, poor CVs were associated with measurements close to the LOD (data not shown). Interbatch CVs were very low, with all detected peptides exhibiting CVs below 10% (Figure 2D). Together, these data prove that the DDA-MS2 quantification method is robust, with quantification of the majority of peptides proving reproducible.

### Quantification of Neuropeptide Proteoforms in CSF from Individuals with Alzheimer’s Disease

165 individuals were divided into four groups on the basis of CSF Amyloid biomarker status^17−20^ and clinical cognitive assessments. Healthy controls (HC, Amyloid negative, no cognitive impairment, *n* = 32), AD-Dementia (AD-DEM, Amyloid positive, dementia, n = 47), AD-MCI (Amyloid positive, mild cognitive impairment, *n* = 63), AD-Asymptomatic (AD-Asymp, Amyloid positive, no cognitive impairment, *n* = 23). The four groups were well matched for age and there were more women in the AD-Asymp group than the other 3 groups (Table 1).

**Figure 3:**
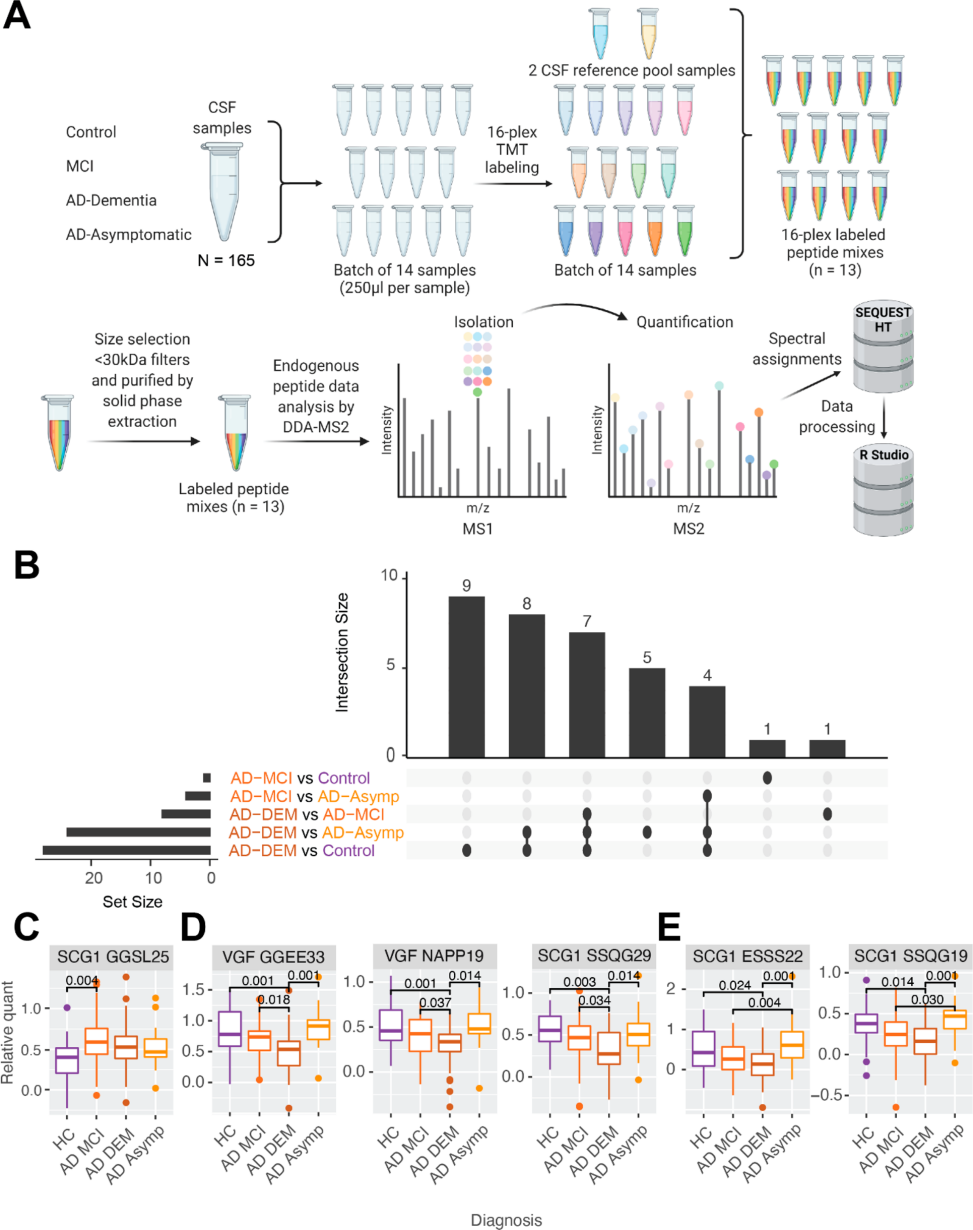
Quantification of neuropeptide proteoforms in CSF from individuals with Alzheimer’s disease. (A) Experimental scheme for neuropeptide quantification in CSF from individuals with Alzheimer’s disease and other diagnostic groups. (B) Upset plot showing the number of neuropeptide proteoforms significantly related to contrasts in experimental groups. (C) SCG1 (GGSL-25) was more abundant in AD-MCI than in healthy controls. (D) Examples of neuropeptide proteoforms significant in the cognitive contrasts including HC versus AD-DEM, AD-MCI versus AD-DEM, and AD-Asymp versus AD-DEM. (E) Examples of neuropeptide proteoforms significant in the cognitive contrasts including HC versus AD-DEM, AD-Asymp versus AD-DEM, and AD-MCI versus AD-Asymp. Significance is denoted by a Benjamini–Hochberg adjusted *p* value below 0.05 from a linear regression which includes age and sex as covariates. Created with BioRender.com.

CSF samples were randomized and analyzed with an inclusion list defined targeted DDA-MS2 method in 13 TMTpro 16 plexes (Figure 3A). Across the whole experiment, 183 peptides were identified from the target neuropeptides. 66 peptides had zero missing values across all 13 plexes, with most missing values occurring in a plex-wise pattern (Supplementary Table 10). Peptides with greater than 20% missing values were excluded from further analysis, leaving 101 peptides for downstream analysis. Across all samples there was a high level of positive correlation both between proteoforms from the same neuropeptide, and between proteoforms from different neuropeptides (Supplementary Figure 2). Multiple proteoforms arising from the same N-terminal protease cleavage event were strongly positively correlated, such as the SCG1 proteoforms, SSQG (SCG1_293-*x*_) and YGEE (SCG1_474-*x*_).

To identify neuropeptide proteoforms significantly associated with cognitive diagnosis, a linear model was fitted with diagnosis, age, and sex as explanatory variables, and *p* values were adjusted using the Benjamini–Hochberg procedure^36^ (Supplementary Table 11). Four SCG1 proteoforms were significantly decreased with age (Supplementary Figure 3A), and 13 from CHGA, SCG-1, -2, -5, and VGF, including VGF_485–503_ (NAPP-19), were significantly lower in men than in women (Supplementary Figure 3B). 35 unique proteoforms from CHGA, SCG-1 and -3, and VGF were associated with at least one diagnostic contrast (Figure 3B, Supplementary Figure 4). All significant proteoforms were most abundant in cognitively unimpaired individuals, except for SCG1_296–320_ (GGSL-25), which was more abundant in AD-MCI than in healthy controls (Figure 3C). Seven proteoforms, including VGF_485–503_ (NAPP-19), were significant in all contrasts against AD-DEM (Figure 3D, Supplementary Figure 4). Four were significantly more abundant in AD-Asymp individuals compared to both AD-MCI and AD-DEM (Figure 3E, Supplementary Figure 4). The GGEE (VGF_373-*x*_) and SSQG (SCG1_293-*x*_) families of proteoforms feature in a number of these significant cognitive contrasts and may be particularly associated with cognitive performance.

Finally, we correlated each neuropeptide proteoform with the Aβ_42_/Aβ_40_ ratio, pTau 181, and total Tau concentrations from the same CSF samples. *p* values were corrected according to the Benjamini–Hochberg procedure, and a surprisingly low number of significant correlations with these analytes was observed (Supplementary Table 12). The majority of significant correlations with these analytes arose from the GGSL (SCG1_296-*x*_) proteoform family. GGSL-15, -17, -19, -22, -23, and -25 all correlate tightly with each other (Supplementary Figure 2) and correlate positively with either pTau 181, total Tau, or both analytes (Supplementary Figure 5A,B). The longest, SCG1_296–320_ (GGSL-25), also correlated negatively with the Aβ_42_/Aβ_40_ ratio (i.e., GGSL-25 increases as disease likelihood increases, Supplementary Figure 5C). VGF_489–503_ (EPVP-15) correlated negatively with pTau 181 (i.e., increases as disease likelihood decreases, Supplementary Figure 5A), while six proteoforms from CHGA correlate positively with total Tau (i.e., increase as disease likelihood increases, Supplementary Figure 5B).

### Mapping and Quantification of Tissue Neuropeptide Proteoforms

**Figure 4:**
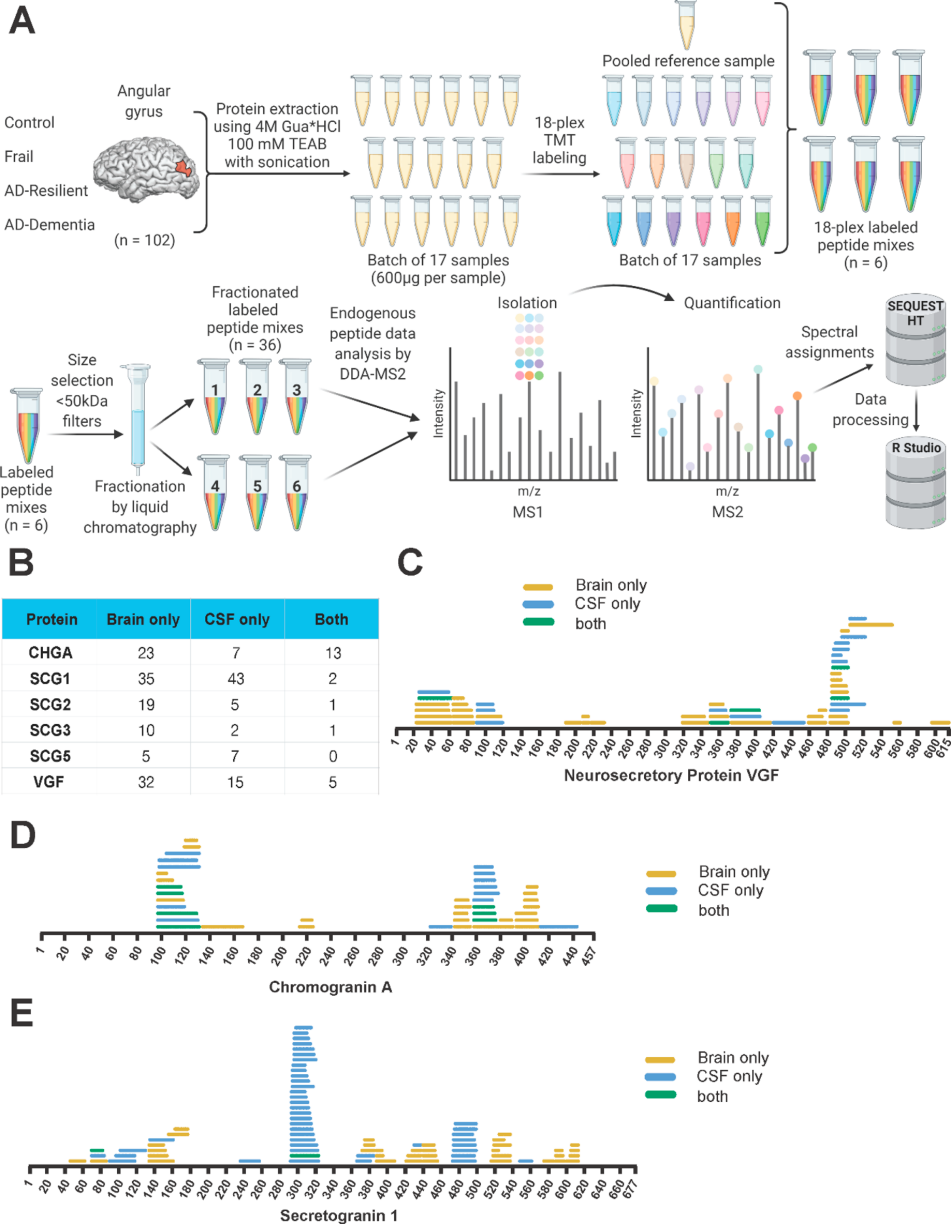
Mapping of tissue neuropeptide proteoforms. (A) Experimental scheme for neuropeptide proteoform quantification in brain tissue from individuals with Alzheimer’s disease and other diagnostic groups. (B) Comparison of neuropeptide proteoforms confidently identified in quantitative experiments in brain only, CSF only, and in both matrices. (C) Mapping of VGF proteoforms, (D) CHGA proteoforms, and (E) SCG1 proteoforms in brain only (yellow), CSF only (blue), and both matrices (green). Created with BioRender.com.

High-resolution neuropeptide proteoform mapping and quantification were performed in a single experiment (Figure 4A) in human post-mortem brain tissue from 102 individuals spanning four diagnostic groups (Table 2) with matched neuropathology but divergent cognitive status: Controls (Braak score ≤4, no cognitive impairment), AD-DEM (Braak score >4, cognitive impairment), AD-Resilient (Braak score >4, no cognitive impairment), and Frail (Braak score ≤4, cognitive impairment). Individual peptide samples were TMT labeled and pooled into six TMTpro 18 plexes (17 samples, one pooled reference per plex). Each TMT plex was split into six analytical fractions by basic reversed-phase fractionation, with each analytical fraction analyzed over a 3 h gradient by MS using a hybrid acquisition scheme which prioritized fragmentation of inclusion list targets while also performing a lower priority regular DDA acquisition scheme. 140 proteoforms from the target neuropeptides were identified in at least one sample, with 128 of these proteoforms having fewer than 20% missing values (Supplementary Table 13). Despite their prioritized acquisition, only 22 of the CSF identified proteoforms were also identified in the brain (Figure 4B). As with CSF, we identified VGF_485–503_ (NAPP-19) and while we did not identify VGF TLQP-21 or TLQP-62, we identified VGF_554–561_ (TLQP-8).

For some brain-identified proteoforms, the CSF proteoforms were clear downstream cleavage products. Brain proteoforms were also identified in regions with no CSF coverage (Figure 4C–E, Supplementary Figure 6). As with the CSF, in general there was a high level of positive correlation between proteoforms from the same protein. Unlike CSF, the correlation between proteoforms from different proteins was generally lower, including a block of VGF proteoforms (including NAPP-19 and TLQP-8) that is strongly inversely correlated with a block of CHGA proteoforms (including AYGF, CHGA_379-*x*_ and GWRP, CHGA_393-*x*_ proteoforms (Supplementary Figure 7).

**Figure 5:**
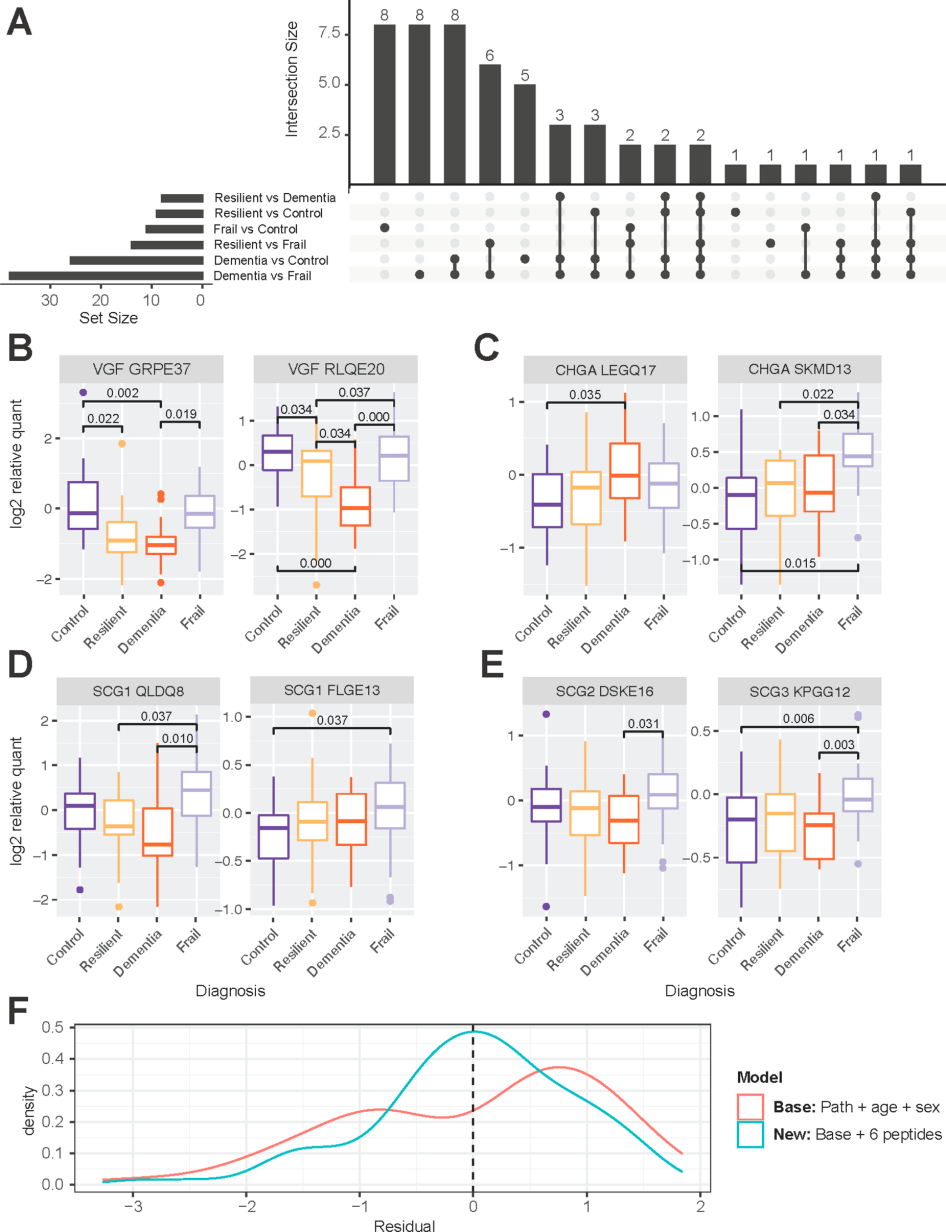
Quantification of tissue neuropeptide proteoforms in individuals with Alzheimer’s disease. (A) Upset plot showing the number of neuropeptide proteoforms significantly associated with between-group contrasts. (B) VGF proteoforms from brain tend to show a similar pattern between groups, with the lowest levels in individuals with AD-DEM. (C) Significant between group associations with CHGA proteoforms show different patterns, with increased levels in AD-DEM versus Controls in LEGQ-17 while the levels were the highest in Frail individuals for SKMD-13. (D) Examples of two SCG1 proteoforms that are highest in Frail individuals. (E) Examples of a SCG2 and SCG3 proteoform that are highest in Frail individuals. Significance is denoted by a Benjamini–Hochberg adjusted *p* value below 0.05 from a linear regression model which includes age and sex as covariates. (F) Adding abundance values of six neuropeptide proteoforms to a linear model predicting global cognition improves the performance of the linear model. When six neuropeptide proteoforms are added to the linear model (blue), residuals are centered around 0 with a more normal distribution than in the base model (red).

Peptides were quantified at the MS2 level. To identify which neuropeptide proteoforms were associated with diagnosis, a simple linear model was fit with diagnostic group, age, and sex as explanatory variables, and *p* values were multiple-test adjusted using the Benjamini–Hochberg procedure (Supplementary Table 14). No proteoforms were significantly associated with age or sex. 53 unique proteoforms, were significantly associated with at least one diagnostic contrast (Figure 5A, Supplementary Figure 8). All but one of the 23 VGF proteoforms were significantly more abundant in Controls and Frail individuals than AD-DEM, suggesting a strong association with AD pathology (Figure 5B, Supplementary Figure 8). Values for these proteoforms in AD-Resilient individuals tended to be intermediate between Controls and those with AD-DEM, with the decrease in AD-Resilient individuals compared to Controls being significant for eight proteoforms (Figure 5B, Supplementary Figure 8).

The non-VGF proteoform targets had more variability in their patterns of abundance across diagnostic groups. In CHGA, some proteoforms were significantly higher in AD-DEM than Controls, and others were significantly higher in Frail individuals than Controls or AD-DEM/Resilient (Figure 5C, Supplementary Figure 8). For SCG1, most significant proteoforms were more abundant in Frail than AD-DEM individuals, with a further four proteoforms more abundant in Frail individuals than Controls (Figure 5D, Supplementary Figure 8). For SCG2 and SCG3, like SCG1, most proteoforms were significantly associated with the AD-DEM versus Frail contrast, with greater abundance in Frail individuals (Figure 5E, Supplementary Figure 8), suggesting a unique mechanism of dysregulation in Frail individuals with a complex relationship to AD pathology.

Given the trend toward intermediate expression of some proteoforms in AD-Resilient individuals compared to Controls and AD-DEM, and the strong relationships of other proteoforms with Frail participants, we sought to ask if addition of proteoform levels to a predictive model of global cognitive score would improve predictions in this population. A base model was fit, which used global pathology score (a summary score encompassing both amyloid and Tau pathology), age at death and sex as explanatory variables:
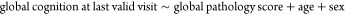
With this base model, approximately 10% of the variation in global cognitive score was explained by these variables. To select peptides to add to this base model, we added peptides with the largest coefficients in an elastic net regression model one by one to the base model until a likelihood ratio test showed that it no longer improved the previous model. The addition of CHGA_342–352_ (WSKM-11), SCG1_375–385_ (PQSE-11), SCG1_388–395_ (NYPS-8), SCG1_604–613_ (VAQL-10), VGF_23–59_ (APPG-37), and VGF_64–79_ (NSEP-16) proteoforms significantly improved the predictive model (*p* < 0.001), increasing the percent explained variability in global cognitive score to 41% and significantly decreasing and normalizing model residuals (Figure 5F). The coefficients of CHGA_342–352_ (WSKM-11), SCG1_375–385_ (PQSE-11), and SCG1_388–395_ (NYPS-8) were negative, meaning that higher levels of these proteoforms were associated with lower cognitive scores (Supplementary Figure 9). For SCG1_604–613_ (VAQL-10), VGF_23–59_ (APPG-37), and VGF_64–79_ (NSEP-16), the association was positive, meaning that higher levels of these proteoforms were associated with higher cognitive scores (Supplementary Figure 9).

### Quantification of Tissue Full-Length Neuropeptide Levels

**Figure 6:**
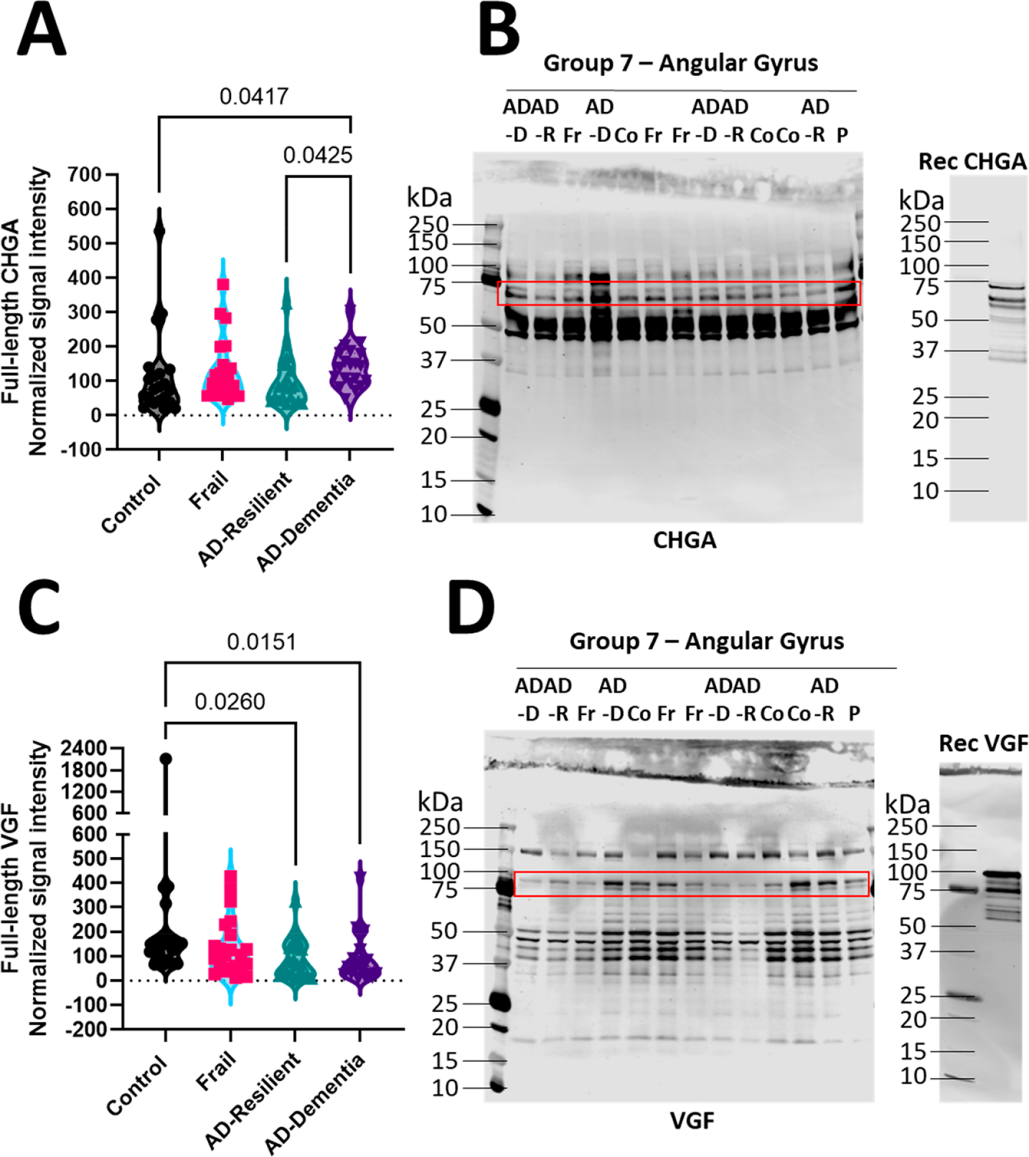
Quantification of tissue full-length neuropeptide levels. (A, C) Semiquantitative densitometry of fluorescence was performed following immunoblotting using LI-COR Image Studio Lite. Data are expressed as the ratio of full-length CHGA (A) and VGF (C) to the pooled sample relative to the amido stain. Data are visualized by violin plot with all points shown, *n* = 84. Data were analyzed using the Kruskal–Wallis test with the Dunn’s test for multiple comparisons. (B, D) Angular Gyrus samples were harvested from the four diagnostic groups, Control (Co, *n* = 21), AD-DEM (AD-D, *n* = 20), AD-Resilient (AD-R, *n* = 21), Frail (Fr, *n* = 22), and the pooled sample (P) were separated by SDS–PAGE (20 μg total protein/lane). Nitrocellulose membranes were immunoblotted with CHGA (B), and VGF (D) specific antibodies then were stained with amido black as a loading control; representative images of the immunoblots are shown. The red band indicates the full-length CHGA and VGF analyzed. Both proteins exist as a doublet that corresponds to the full-length protein with and without the signal peptide as shown by the recombinant proteins run on a separate immunoblot but probed with the same antibody, recombinant chromogranin A was loaded at 22 ng and recombinant VGF was loaded at 15 ng per lane. Significant values are shown with a *p* value of less than 0.05.

As the nontryptic proteomics could not quantify full-length neuropeptide proteins, immunoblotting was used (Figure 6, Supplementary Figures 10 and 11). Full-length CHGA was significantly increased in AD-DEM relative to Control and AD-Resilient individuals (Figure 6A,B). Full-length VGF was significantly decreased in both AD-Resilient and AD-DEM relative to Controls (Figure 6C). Lower molecular weight bands were detected with both antibodies that likely reflect forms that have undergone post-translational modifications including proteolysis. No significant differences were observed in full-length secretogranins, SCG-1, -2, -3, and -5 (Supplementary Figure 10A,C,E,G). All unedited immunoblots alongside their respective amido black stains are detailed in Supplementary Figure 11.

### Identification of Novel Proteases Able to Cleave Neuropeptides

**Figure 7:**
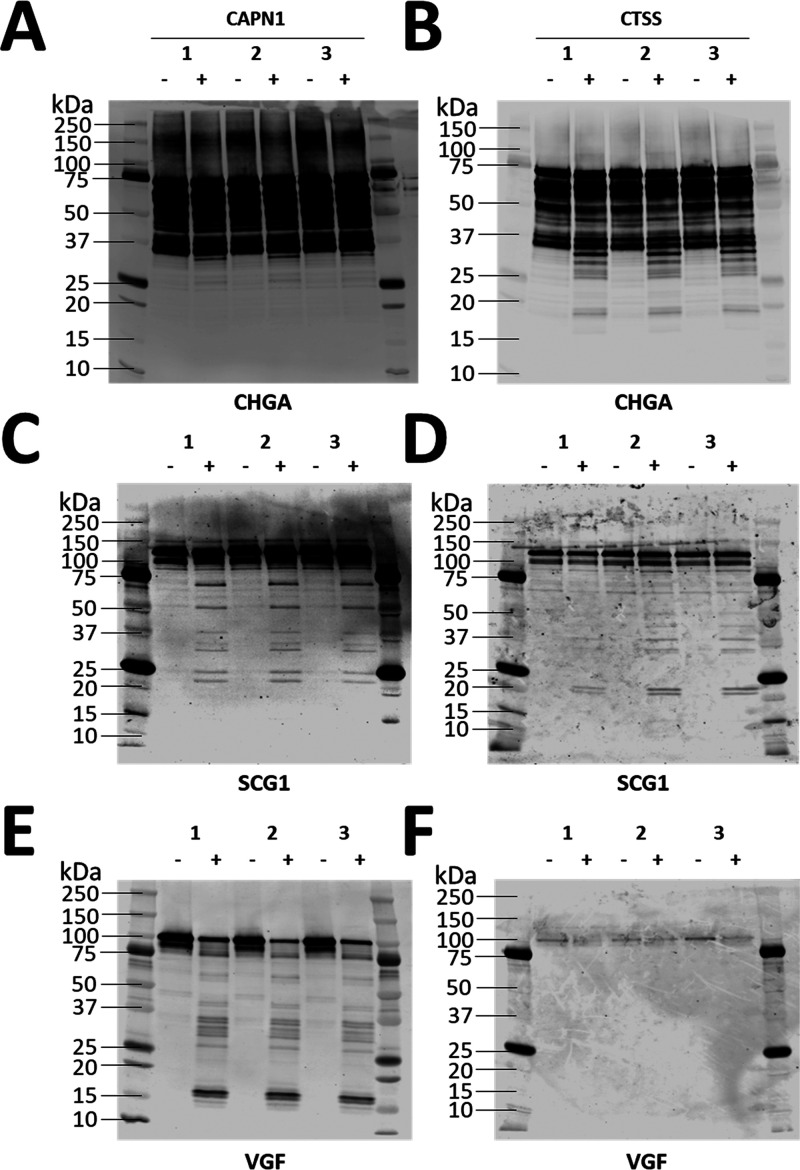
Recombinant protease digestion of CHGA, SCG1, and VGF by Calpain-1 and Cathepsin S. 1 μg of recombinant human CHGA (A, B), SCG1 (C, D), and VGF (E, F) were incubated with 20 ng of recombinant human CAPN1 at 30 °C for 10 min (A, C, E) or 10 ng of recombinant human CTSS at 37 °C for 1 h (B, D, F). All digestions were performed in triplicate, reactions were stopped by freezing at −80 °C, and 1 μL of the total reaction volume was diluted in 1× loading buffer and heated at 95 °C for 5 min for immunoblot analysis. Nitrocellulose membranes were immunoblotted with CHGA (A, B), SCG1 (C, D), and VGF (E, F) specific antibodies. Amido black was not used as a loading control due to the low amount of protein that was separated.

**Figure 8:**
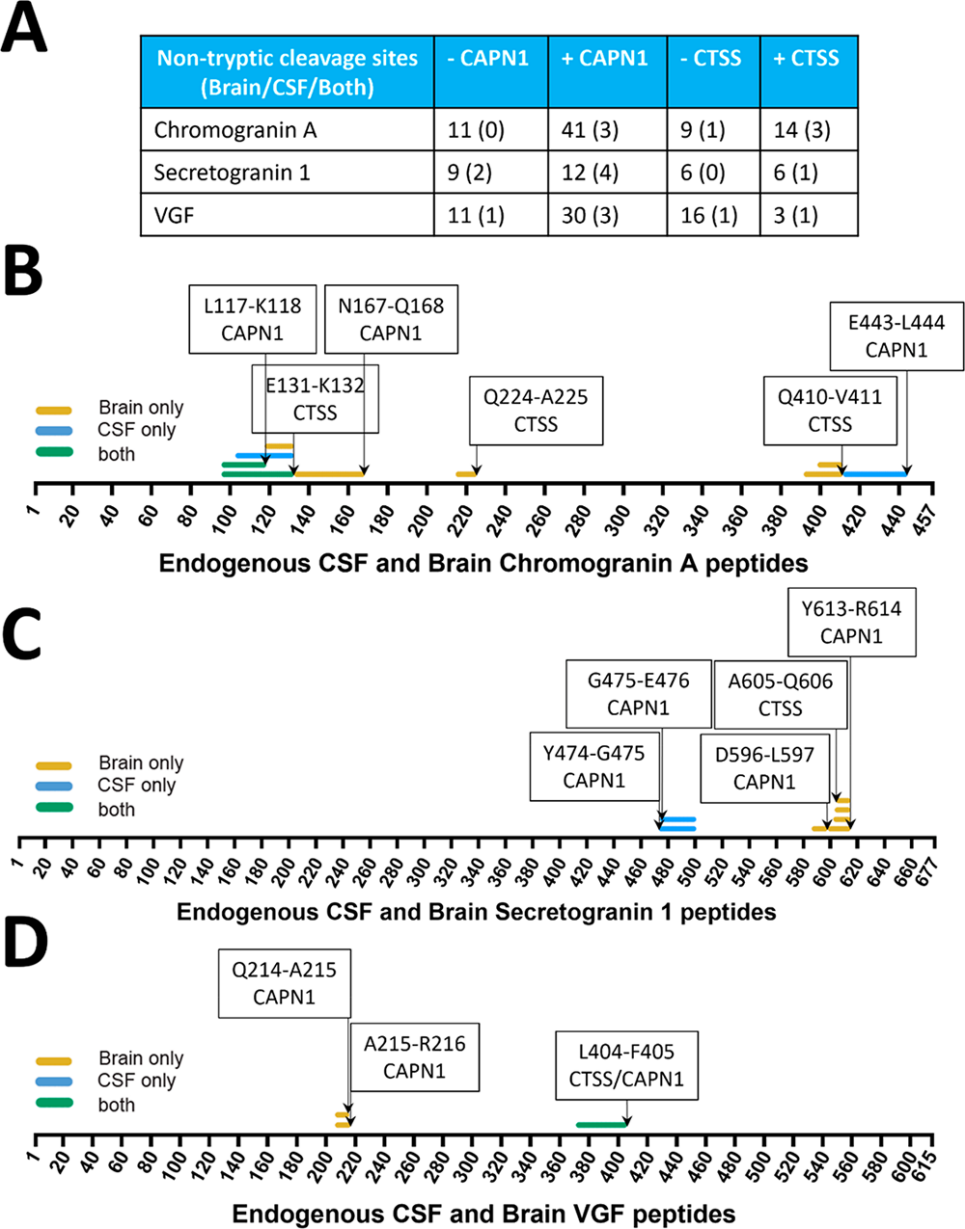
Identification of Calpain-1 and Cathepsin S cleavage sites within CHGA, SCG1, and VGF. Recombinant protein digestions combining CHGA, SCG1, VGF, and Calpain-1 or Cathepsin S were performed as described in Figure 7. Recombinant protein digestions were subject to trypsin digestion and subsequent MS; a nontryptic search was used to identify novel Calpain-1 and Cathepsin S cleavage sites. (A) Nontryptic Calpain-1 and Cathepsin S cleavage sites identified are listed and in parentheses are those that match nontryptic cleavage sites identified in the endogenous neuropeptide proteoforms from brain (yellow), CSF (blue), or both (green). Novel Calpain-1 and Cathepsin S cleavage sites within endogenous brain, CSF, or both neuropeptide proteoforms from Chromogranin A (B), Secretogranin 1 (C), and VGF (D) are highlighted.

All LC–MS/MS-identified CSF and brain neuropeptide proteoforms were uploaded to the PROTEASIX^37^ (Ver Jan 2017) online peptide-centric prediction tool. This tool enables the prediction of potential proteases that cleave at the N- or C-terminus of your peptide of interest from the MEROPS database.^38^ By cross referencing PROTEASIX results with protease brain expression values (human protein atlas^39^ Ver 21), two brain-expressed proteases that could potentially cleave neuropeptides were identified, Cathepsin S and Calpain-1 (Supplementary Table 15). Recombinant human CHGA, SCG1, and VGF were incubated with or without Calpain-1 for 10 min at 30 °C or Cathepsin S for 1 h at 37 °C in triplicate and then subjected to immunoblot analysis using neuropeptide-specific antibodies (Figure 7) and LC–MS/MS analysis (Figure 8). Calpain-1 cleaved CHGA to produce bands that migrated at ∼35 kDa (Figure 7A) while Cathepsin S cleaved CHGA to produce multiple bands that migrated between 18 and 35 kDa (Figure 7B). The additional bands between 100 and 250 kDa (Figure 7A) represent an increased exposure time in comparison to the CTSS experiment (Figure 7B). Calpain-1 cleaved SCG1 to produce multiple bands that migrated at 20, 25, 30, 37, 50, and 70 kDa (Figure 7C), while Cathepsin S cleaved SCG1 to produce multiple bands that migrated at 18, 20, 35, 37, and 50 kDa (Figure 7D). Calpain-1 cleaved VGF to produce multiple bands that migrated between 14 and 70 kDa (Figure 7E) while Cathepsin S cleaved VGF as shown by a small visual reduction in the amount of full-length VGF; however, no cleaved bands of VGF were identified using this antibody (Figure 7F). It is important to note that endogenous degradation in the -CAPN1 and -CTSS groups was observed (Figure 7).

LC–MS/MS was used to identify Calpain-1 and Cathepsin S cleavage sites within CHGA, SCG1, and VGF (Figure 8). Samples were trypsin digested and a nontryptic search performed to enable the identification of cleavage sites due to Calpain-1 and Cathepsin S (Figure 8A; Supplementary Table 16). Novel nontryptic Calpain-1 and Cathepsin S cleavage sites of CHGA, SCG1, and VGF were then cross referenced to the nontryptic cleavage sites identified in brain and CSF samples (Figure 8A; Supplementary Table 16). For chromogranin A, 41 nontryptic cleavage sites were identified in the + Calpain-1 group compared to 11 in the – Calpain-1 group (Figure 8A). Three nontryptic cleavage sites identified in the + Calpain-1 digest were observed in the brain and CSF analyses, L117-K118 (CHGA_97–117_; HSGF-21), N167-Q168 (CHGA_134–167_; EDSK-34), E443-L444 (CHGA_413–443_; GYPE-31), while none were identified in the – Calpain-1 digest (Figure 8A). These novel nontryptic cleavage sites were mapped (Figure 8B, C, D). We found four novel Calpain-1 and one Cathepsin S cleavage sites of Secretogranin 1, while two novel Calpain-1 and one of both Cathepsin S and Calpain-1 cleavage sites of VGF were identified (Figure 8D). No significant between-group differences were observed in Cathepsin S nor Calpain-1 levels in brain tissue (Supplementary Figure 12). All unedited immunoblots alongside their respective amido black stains are detailed in Supplementary Figure 13.

## Discussion

The aim of this work was to improve our understanding of granin neuropeptide diversity, regulatory mechanisms, and their association with cognitive impairment in AD-Dementia. We developed a novel MS assay to identify and quantify endogenous nontryptic neuropeptide proteoforms in the CSF and brain tissue from subjects with matched levels of neuropathology but diverse cognitive performance. We studied the levels of full-length neuropeptides in brain tissue from this sample set using immunoblots. We predicted potential proteases involved in the proteolytic cleavage of neuropeptides and studied whether any of these predicted proteases were able to cleave neuropeptides in vitro.

Our study built on previous peptidomics work,^6^ as most of the literature has focused on using tryptic digestion upstream of analysis via MS.^1^ When samples are trypsin digested, it becomes difficult to distinguish whether an identified peptide arises from the full-length protein or an endogenously protease-cleaved peptide proteoform. Nontryptic peptidomics removes the full-length protein from the analyzed mixture, enabling confident identification of endogenously cleaved peptides. The disadvantage of this technique is that any amino acid can be considered a potential cleavage site, which makes spectral identification more complex, and more are subject to loss from multiple tests. To minimize these issues, we used two approaches to identify spectra, the standard Proteome Discoverer mapping and PEAKS “sequencing approach”. We then ran multiple rounds of acquisition and technical optimization to ensure identified peptides were robust. This approach enabled us to map and quantify many novel proteoforms from the target neuropeptides, increasing our knowledge of the complexity of these proteins. To compare the performance of our assay to a tryptic approach, we performed a semitryptic MaxQuant search of our previous fractionated data from the human dorsolateral prefrontal cortex^40^ and mined this data for novel protease cleavage sites in the neuropeptides of interest. Of the 66,000 identified peptides in this search, 89.4% have a tryptic cleavage site at the N-terminal and 94.1% have a tryptic cleavage site at the C-terminal. We identified zero VGF peptides with nontryptic ends and two peptides from CHGA with nontryptic ends. Of these 2 tryptic CHGA peptides, we identify EAVEEPSSKDVME, which has the C-terminal cleavage site common to HSGF-35, SGFE-34, LSEV-28, and EAVE-13, and SGFEDELSEVLENQSSQAELK, which contains the common N-terminal cleavage site identified in SGFE-34 and -35. Both these sites may arise from the same initial cleavage events, as our data shows they are found at opposite ends of the same longer peptide, SGFE-34. Due to the tryptic cleavage sites in the middle of the peptide, this information is not possible to obtain from the tryptic data. We therefore believe that our enrichment method enables identification of a more diverse range of peptide products and better assemblage of intact endogenous proteoforms and has more sensitivity to lower abundance forms than a semitryptic search.

Neuropeptide proteoforms may have different functions compared to the full-length protein. Full-length VGF is synthesized in neurons and neuroendocrine cells, where it has roles in regulating energy balance, metabolism, learning and memory, synaptogenesis, and neurogenesis.^1,14−16^ However, specific VGF proteoforms have been shown to have differential effects on AD pathogenesis;^1^ for example, VGF_554–615_ (TLQP-62) binds neurons to regulate long-term memory formation and prevent memory deficits in mice^41^ while VGF_554–574_ (TLQP-21) binds microglia leading to microglial chemotaxis and phagocytosis resulting in a reduction in Aβ plaques and decreased neuritic dystrophy in mice.^42,43^ Of note, we did not detect either TLQP-21 or TLQP-62 in the brain or CSF of patients with AD; however, we detected VGF_554–561_ (TLQP-8) in the brain samples. While TLQP-62 is likely outside of the detection range of the mass-spectrometer, in theory TLQP-21 should be detectable. Unfortunately, while a significant identification of a peptide is usually meaningful in LC–MS/MS, a lack of detection could be due to technical reasons (ion interference from a more abundant peptide, for example), and so we cannot draw a solid conclusion as to the presence of TLQP-21. However, TLQP-8 was significantly decreased in AD-DEM compared to AD-Resilient, Frail individuals and Controls (Supplementary Table 14), so may have similar functions to the longer peptides related to both cognitive and pathological features in Alzheimer’s disease.

Our data showed that neuropeptide proteoforms were reliably detected in both CSF and brain tissue. Many of the proteoforms identified in the CSF were likely downstream products of the brain proteoforms. This was highlighted by SCG1. Here, SCG1_293–323_ was detected in both the brain and CSF; however, 12 additional SCG1 peptides starting at amino acid 293 with degradation at the C-terminus were identified in the CSF ranging from SCG1_293–308_ to SCG1_293–322_. This likely represents neuropeptide proteoforms moving from the brain into the CSF and their subsequent degradation. This has been previously identified in other proteins linked to Alzheimer’s disease including Tau,^44,45^ but the mechanism by which this happens is unclear. Given the low overlap in peptides detected in both matrices, there is not much data to suggest if proteoforms with significant differences in brain maintain these differences in CSF. APPE-18, GLQE-21, GRPE-37, and GGEE-32 of VGF are significantly lower in AD-DEM compared to controls in both brain and CSF, but their associations with the other groups are less consistent between matrices. VGF_485–503_ (NAPP-19) is the most well-established VGF peptide that we detected in our study and maintains significant differences between AD-DEM and resilient/asymptomatic individuals in both matrices. One peptide that falls within NAPP-19, VGF_489–503_ (EPVP-15) correlated negatively with pTau 181, the best ATN marker of cognitive progression. NAPP-19 has previously been shown to be decreased in the CSF of patients with Dementia with Lewy Bodies compared to AD or controls^46^ and was decreased in the CSF of AD patients compared to controls.^6^

It is currently unclear whether neuropeptide levels change as a result of transcriptional regulation or differential activity of protease enzymes in disease. We identified both Calpain-1 and Cathepsin S as potential protease enzymes involved in neuropeptide cleavage. Many of the potential proteases we identified using MEROPS were not expressed in the brain and thus were discounted from analysis. Calpain-1 had shown its ability to cleave Tau in the pathogenesis of AD,^47^ and an antibody against active Calpain-1 showed that it increases with Braak stage in AD.^48^ Cathepsin S is able to cleave Tau oligomers in dementia^49^ and has been shown to increase in both Alzheimer’s and Down Syndrome disease brains.^50^ Cathepsins are detected in CSF, but no clear differences have been observed in AD-DEM versus controls in published or our own unpublished CSF data sets.^51^ In our study, we quantified full-length levels of both Calpain-1 and Cathepsin S in the angular gyrus of the different diagnostic groups examined; however, no significant differences were observed in either protease. The abundance of a protease enzyme does not necessarily reflect activity levels, which are further regulated by modification and localization. We further found that full-length CHGA and VGF were significantly differentially abundant between diagnostic groups, while no changes were observed in full-length secretogranins. This is largely in agreement with the endogenous neuropeptide proteoform data from these two proteins, and correlation between neuropeptide levels from the same protein parent was generally very high. Taken together, this data suggest that major regulation of neuropeptide levels in AD is not due to altered levels of proteases but may be due to upstream changes. For VGF, these changes may arise from differences in transcription of the neuropeptide parent gene.^41,52^ Publicly available bulk RNAseq and proteomic data from the same ROSMAP cohort as our samples suggests VGF levels are highest in controls, lowest in AD-DEM, and intermediate in AD-RES in prefrontal cortex tissue (*n* = 626).^53^ CHGA transcript levels are not different between groups in the same data set, and so the mechanism of upregulation in AD tissue is unclear,^53^ Single-cell RNaseq showed VGF was decreased at the transcript level in the entorhinal cortex of AD brains compared to controls.^52^ Boosting VGF at the transcript level in a mouse model of dementia was also shown to have a rescue effect on memory and pathological features of amyloid.^41^

One of the key strengths of our study is the size and the well-characterized nature of our sample cohorts. One caveat of our work is that only the angular gyrus was examined; further studies should utilize different brain regions for MS analysis. We identified that both Cathepsin S and Calpain-1 were able to cleave the neuropeptides, CHGA, SCG1, and VGF into proteoforms that were also identified in the CSF and brains of the different diagnostic groups examined. We showed this using in vitro recombinant protein digestion assays and validated it with both immunoblot and MS analysis. This methodology is unique and highlights the potential of using endogenous peptide information combined with prediction software and digestion assays to identify new cleavage sites within these proteins. Further work is required to validate these cleavage sites utilizing protease inhibitors and cleavage-site mutations in cell and animal models of the disease.

## Conclusion

Using a validated MS assay across two sample matrices, we showed that neuropeptides provide extra information about the cognitive status of an individual, above the information given by their pathology status. As previous literature has also suggested, VGF NAPP-19 is perhaps the strongest candidate for a biomarker of cognitive status, maintaining significant differences between AD-DEM and control and resilient individuals across both brain and CSF. NAPP-19 may be particularly useful as a novel biomarker of synaptic health, cognitive decline, and AD pathology. While peptides from VGF are generally decreased in AD-DEM, peptides from CHGA showed an opposite pattern with some peptides showing increases in AD-DEM and Frail individuals compared to Controls in brain. High levels of within protein correlation, and a lack of abundance differences of novel protease enzymes, suggests that primary regulation of VGF and CHGA in the brain may be transcriptional, and not at the protease level. Future therapeutics may therefore be best targeted at regulation of transcription, as opposed to treatment with individual peptides. Further work should be focused on comparing the cellular effects of peptide treatment compared with transcriptional upregulation.

## Supporting Information Available

Supplementary Figure 1: Comparison of fractionated and unfractionated CSF analysis of secretogranin proteoforms. Supplementary Figure 2: Correlation plot of CSF neuropeptide proteoforms. Supplementary Figure 3: Correlation of CSF neuropeptide proteoforms significantly associated with age and sex. Supplementary Figure 4: All CSF neuropeptide proteoforms that are significantly associated with at least one between group contrast. Supplementary Figure 5: Correlation of CSF neuropeptide proteoforms with ATN values. Supplementary Figure 6: Comparison of brain and CSF neuropeptide proteoforms for secretogranins. Supplementary Figure 7: Correlation plot of brain neuropeptide proteoforms. Supplementary Figure 8: All brain neuropeptide proteoforms that are significantly associated with at least one between group contrast. Supplementary Figure 9: Correlation of brain neuropeptide proteoforms with cognition. Supplementary Figure 10: Quantification of tissue full-length secretogranin levels. Supplementary Figure 11: All immunoblots and amido black stains from brain neuropeptide analysis. Supplementary Figure 12: Quantification of tissue full-length protease levels. Semiquantitative densitometry of fluorescence was performed following immunoblotting using LI-COR Image Studio Lite (PDF)
Supplementary Figure 13: All immunoblots and amido black stains from brain protease analysis (PDF)
Supplementary Table 1: Sample by sample demographic data for the CSF experiment including diagnostic classification, age, sex, and ATN values (XLSX)
Supplementary Table 2: Sample by sample demographic data for the individual brain samples (XLSX)
Supplementary Table 3: TMT 16-plex labeling plan for CSF samples from the different diagnostic groups (XLSX)
Supplementary Table 4: TMT 18plex labeling plan for brain samples from the different diagnostic groups (XLSX)
Supplementary Table 5: Antibodies used in the immunoblot analysis experiments (XLSX)
Supplementary Table 6: Multitab peptide table from fractionated versus unfractionated mass-spectrometry experiment (XLSX)
Supplementary Table 7: Summary table of linearity and limit of detection data by peptide (XLSX)
Supplementary Table 8: Raw data table from reproducibility experiment (XLSX)
Supplementary Table 9: Summary table of peptide-by-peptide performance statistics (XLSX)
Supplementary Table 10: Peptide quantification table from CSF quantification experiment (XLSX)
Supplementary Table 11: CSF regression results (XLSX)
Supplementary Table 12: CSF peptide correlations with ATN values (XLSX)
Supplementary Table 13: Peptide quantification table from brain quantification experiment (XLSX)
Supplementary Table 14: Brain linear regression results (XLSX)
Supplementary Table 15: Identification of proteases that can cleave neuropeptides into the neuropeptide proteoforms identified in the CSF and brain alongside the protein and RNA expression levels of proteases in the brain (XLSX)
Supplementary Table 16: Identification of Calpain-1 and Cathepsin S cleavage sites within CHGA, SCG1 and VGF from recombinant protein digestion assays that correspond to neuropeptide proteoforms identified in the CSF and brain (XLSX)
