# Correction to “Cerebrospinal Fluid and Brain Proteoforms of the Granin Neuropeptide Family in Alzheimer’s Disease”

**DOI:** 10.1021/jasms.3c00265

**Published:** 2023-08-15

**Authors:** James P. Quinn, Elizabeth C. Ethier, Angelo Novielli, Aygul Malone, Christopher E. Ramirez, Lauren Salloum, Bianca A. Trombetta, Pia Kivisäkk, Michael Bremang, Stefan Selzer, Marjorie Fournier, Sudeshna Das, Yaoyi Xing, Steven E. Arnold, Becky C. Carlyle

**Affiliations:** † Massachusetts General Hospital Department of Neurology, Harvard Medical School, Boston, Massachusetts 02129, United States; ‡ Advanced Proteomics Facility, Department of Biochemistry, University of Oxford, Oxford, Oxfordshire OX1 3QU, United Kingdom; § Proteome Sciences LLC, Frankfurt am Main, Hessen 60438, Germany; ∥ Department of Physiology, Anatomy & Genetics, University of Oxford, Oxford, Oxfordshire OX1 3QU, United Kingdom; ⊥ Kavli Institute for Nanoscience Discovery, University of Oxford, Oxford OX1 3QU, United Kingdom

All authors have agreed to the following requested changes:

In the Author Contributions, Yaoyi Xing’s name was misspelled as “Xaoyi Xing.” The corrected paragraph is included in this Correction.

The PRIDE public repository details for the raw files for this work were omitted from the original paper. The raw files can be found at http://www.ebi.ac.uk/pride/archive/projects/PXD037367.

## Data Availability

The PRIDE public repository details for the raw files for this work can be found at http://www.ebi.ac.uk/pride/archive/projects/PXD037367.

