# Supplementary material for: Cerebrospinal Fluid and Brain Proteoforms of the Granin Neuropeptide Family in Alzheimer’s Disease
Source: J Am Soc Mass Spectrom. 2023 Mar 13;34(4):649–67. doi: 10.1021/jasms.2c00341 (PMC10080684; doi:10.1021/jasms.2c00341)
Supplement: Supplementary file 2 — js2c00341_si_002.pdf [file js2c00341_si_002.pdf]

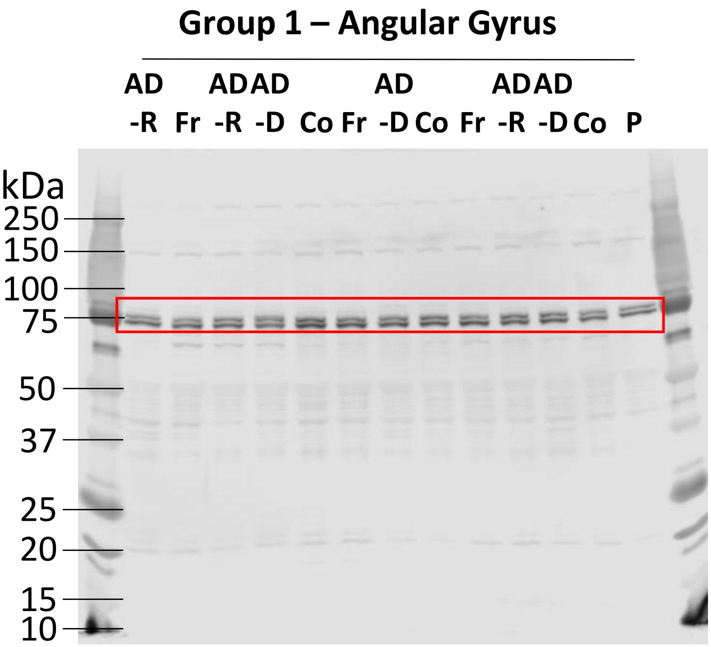

CAPN1

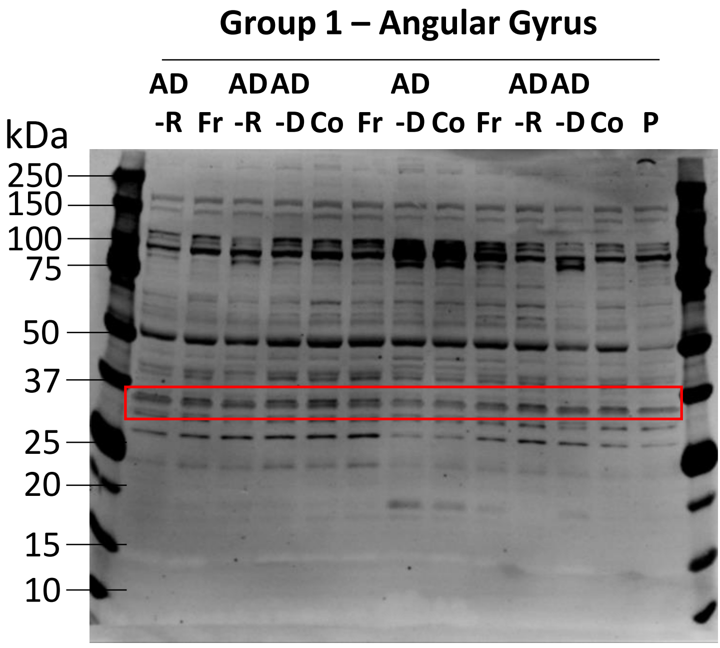

CTSS

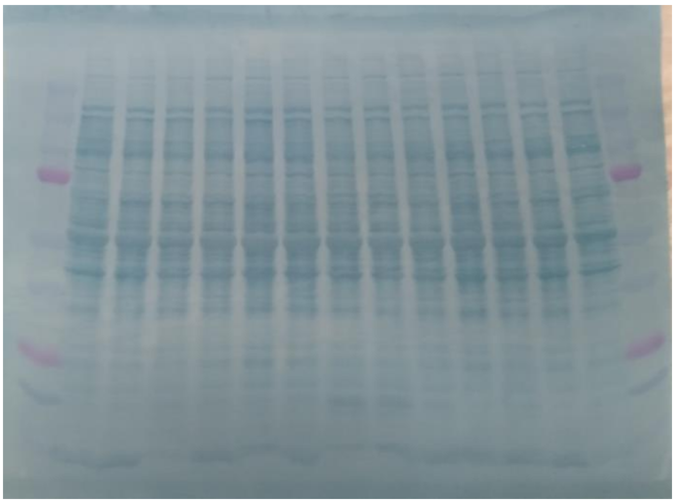

CAPN1 Amido Black

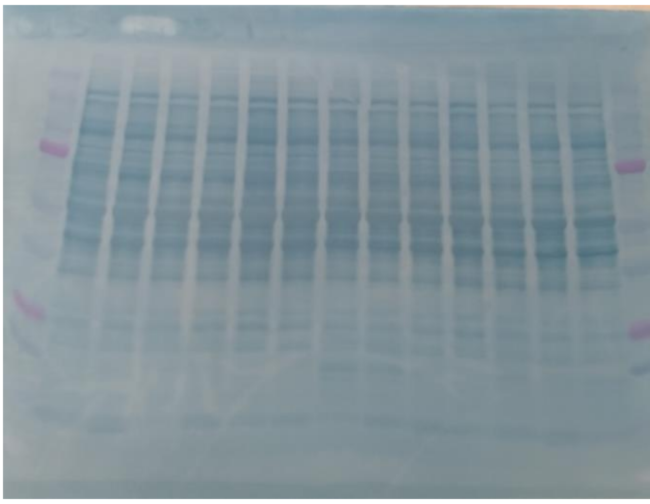

CTSS Amido Black

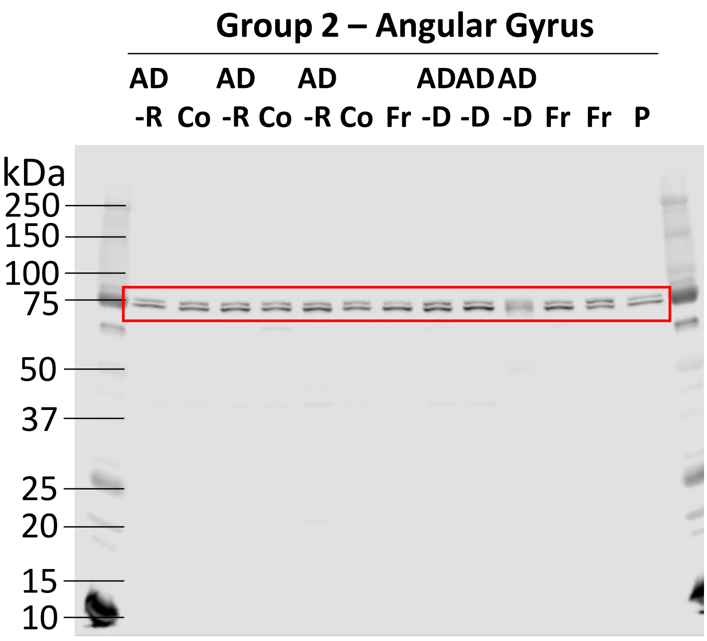

CAPN1

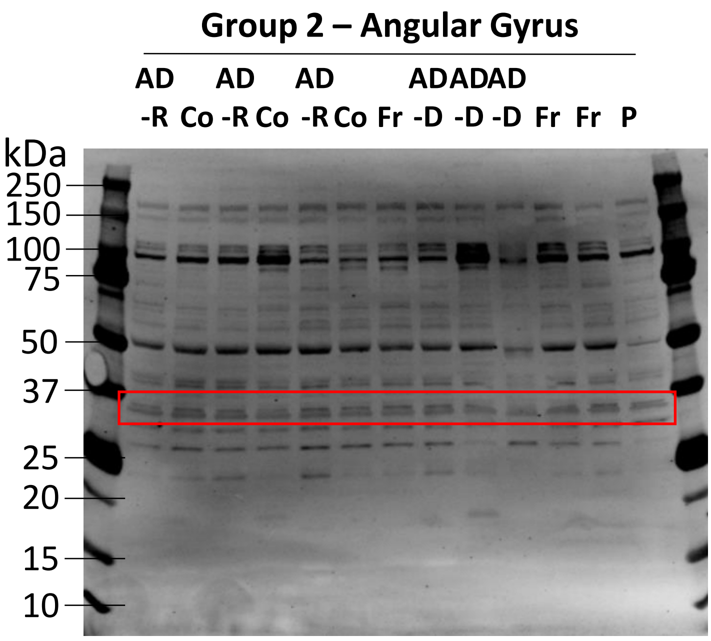

CTSS

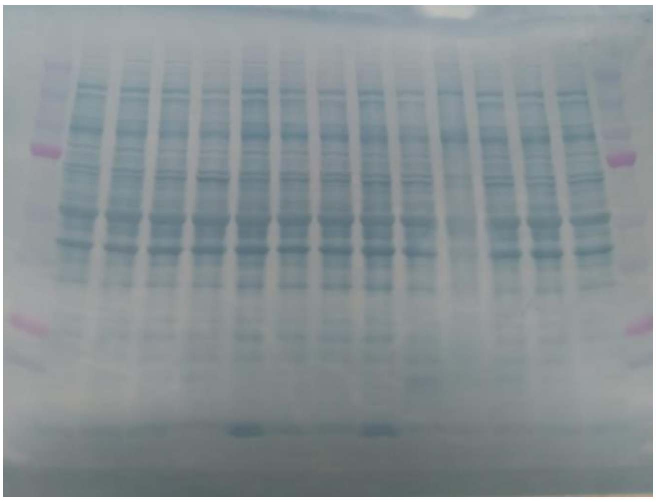

CAPN1 Amido Black

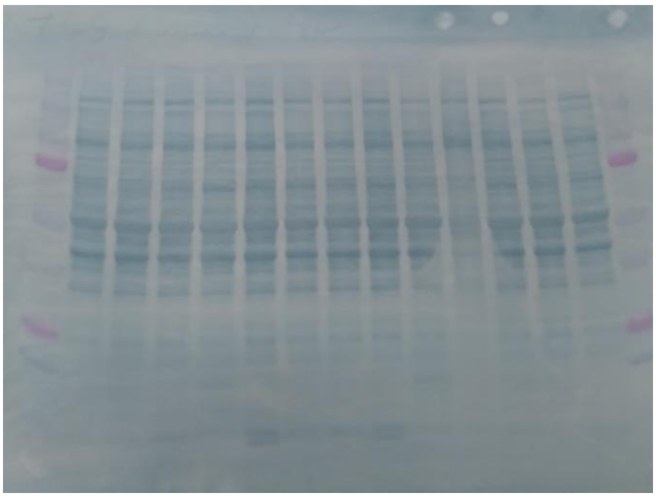

CTSS Amido Black

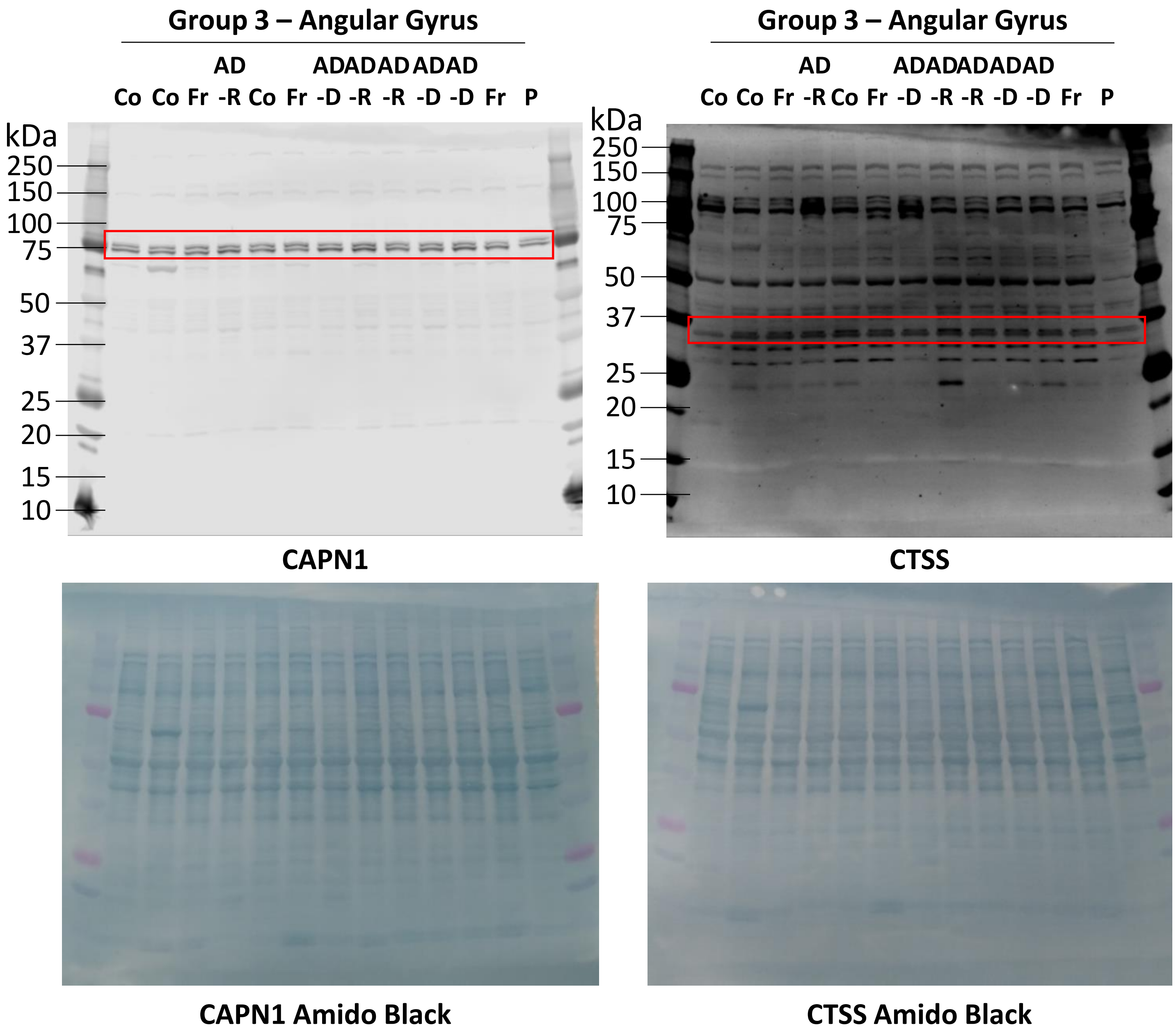

Group 3 – Angular Gyrus
AD ADADADADAD
Co Co Fr -R Co Fr -D -R -R -D -D Fr P
kDa
250
150
100
75
50
37
25
20
15
10
CAPN1
Group 3 – Angular Gyrus
AD ADADADADAD
Co Co Fr -R Co Fr -D -R -R -D -D Fr P
kDa
250
150
100
75
50
37
25
20
15
10
CTSS
CAPN1 Amido Black
CTSS Amido Black

Group 4 – Angular Gyrus

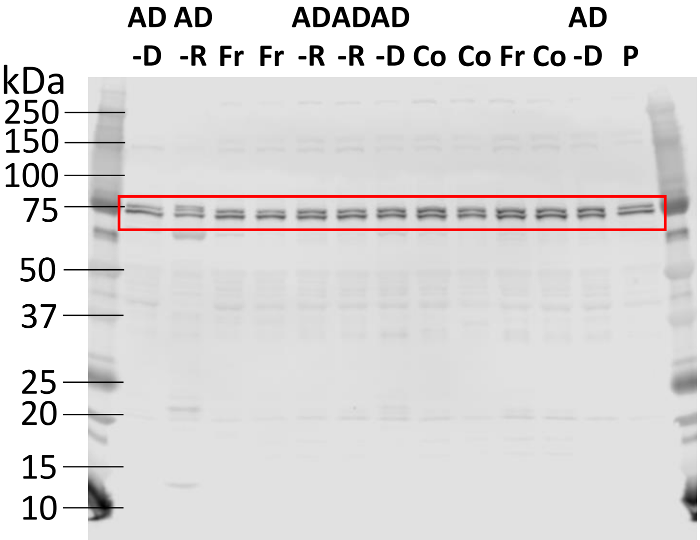

CAPN1

Group 4 – Angular Gyrus

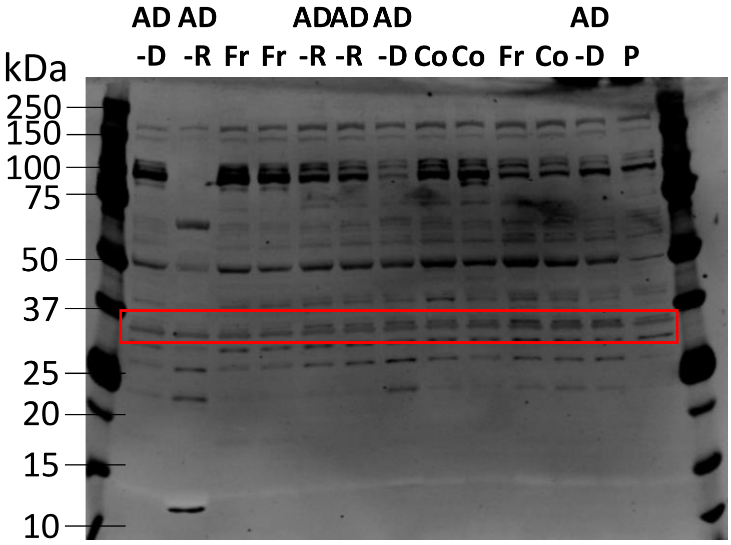

CTSS

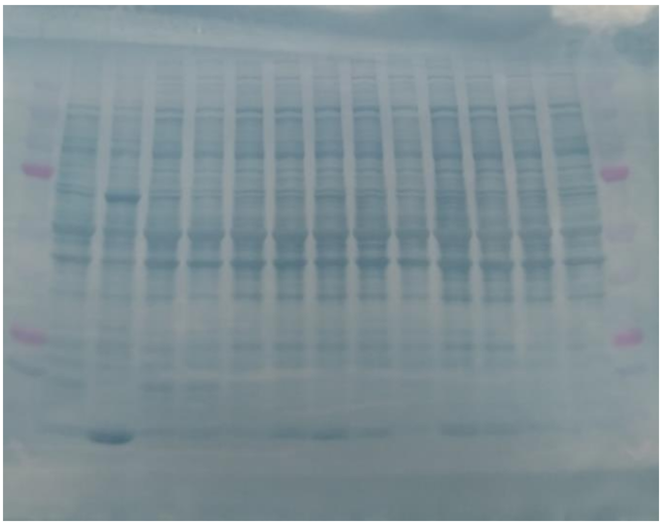

CAPN1 Amido Black

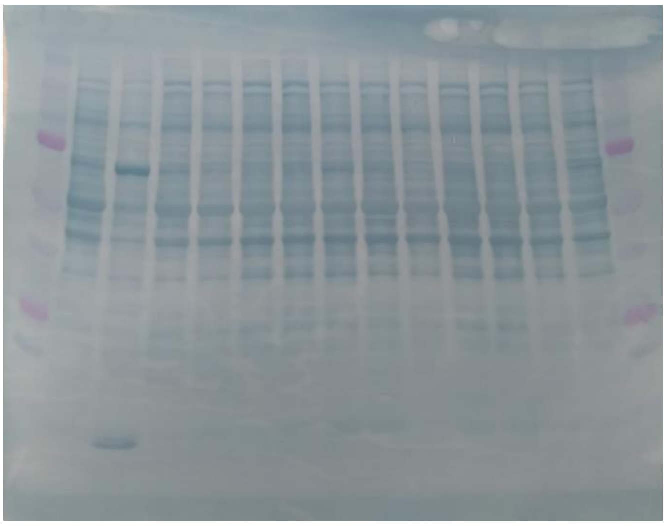

CTSS Amido Black

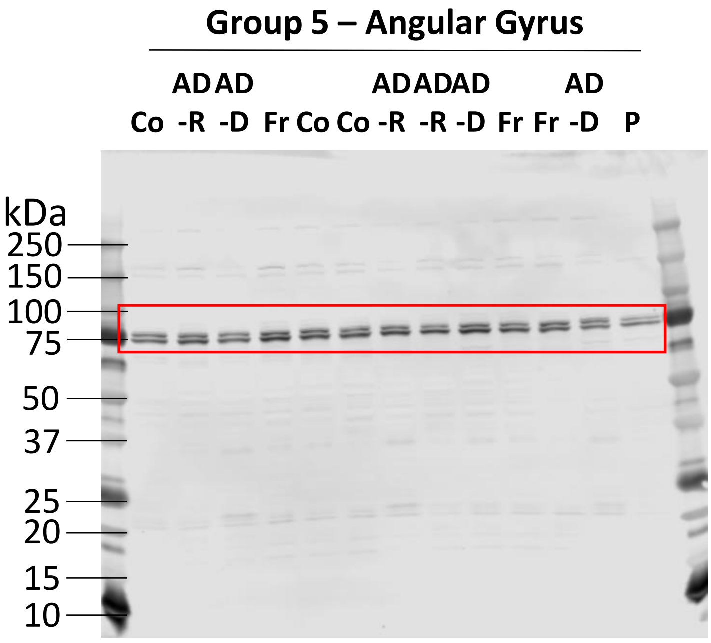

CAPN1

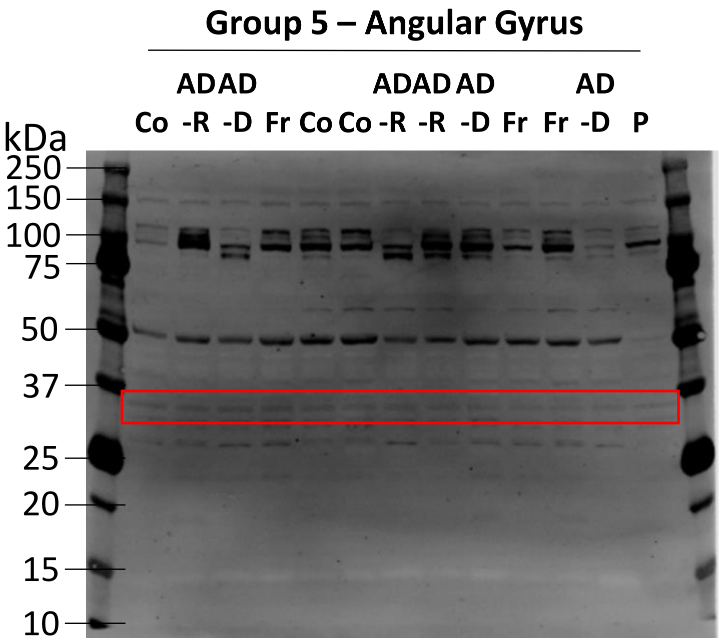

CTSS

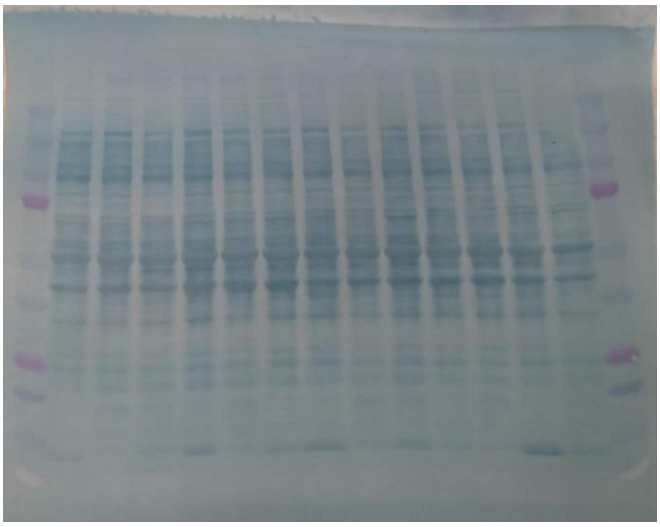

CAPN1 Amido Black

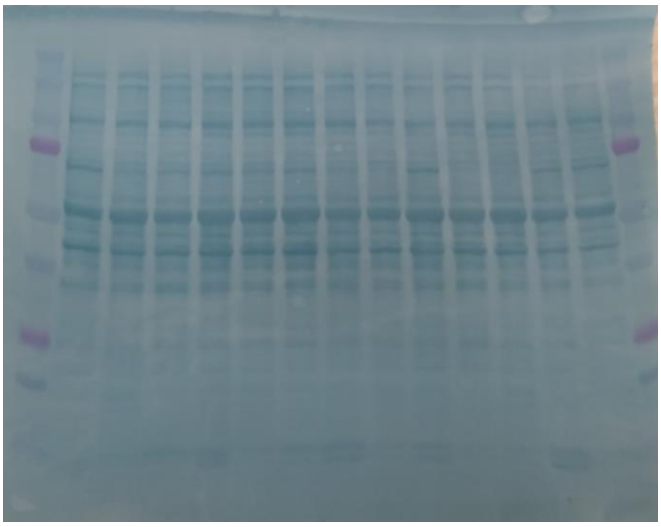

CTSS Amido Black

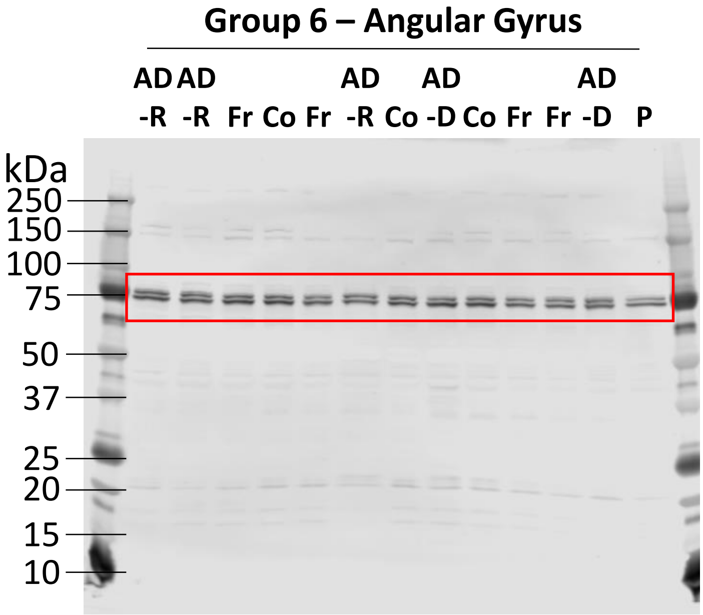

CAPN1

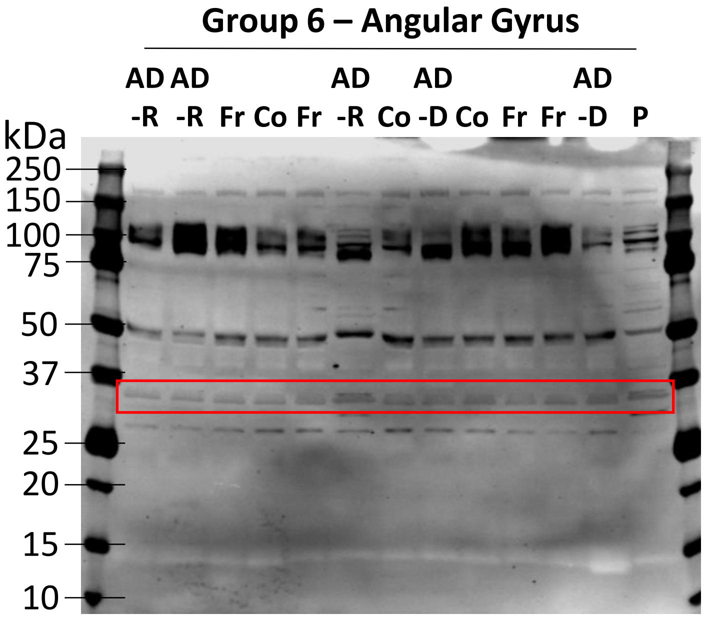

CTSS

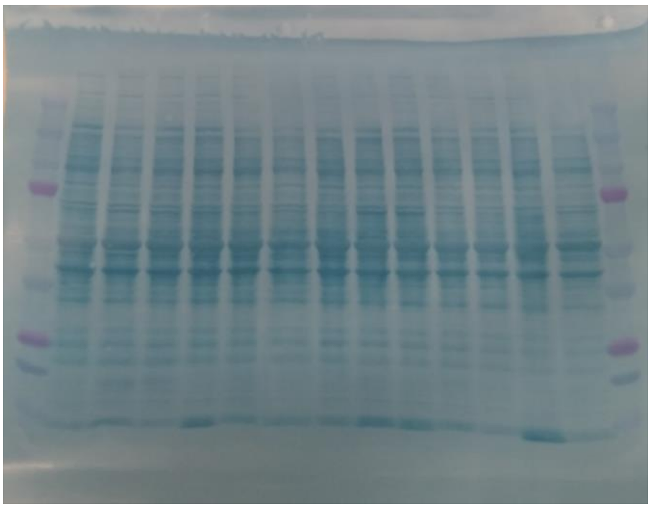

CAPN1 Amido Black

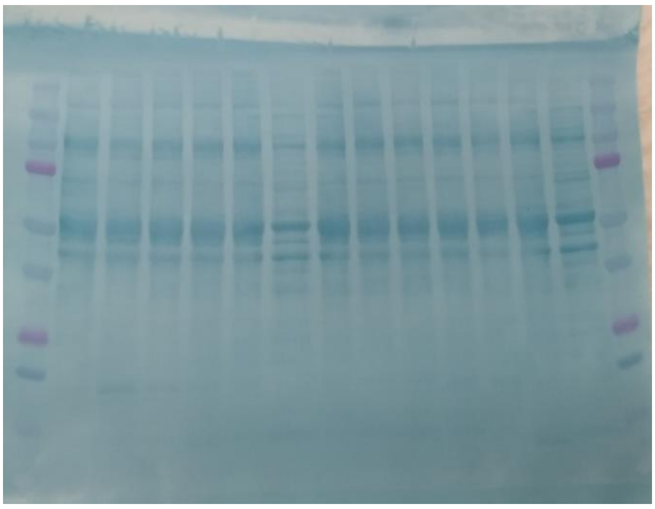

CTSS Amido Black

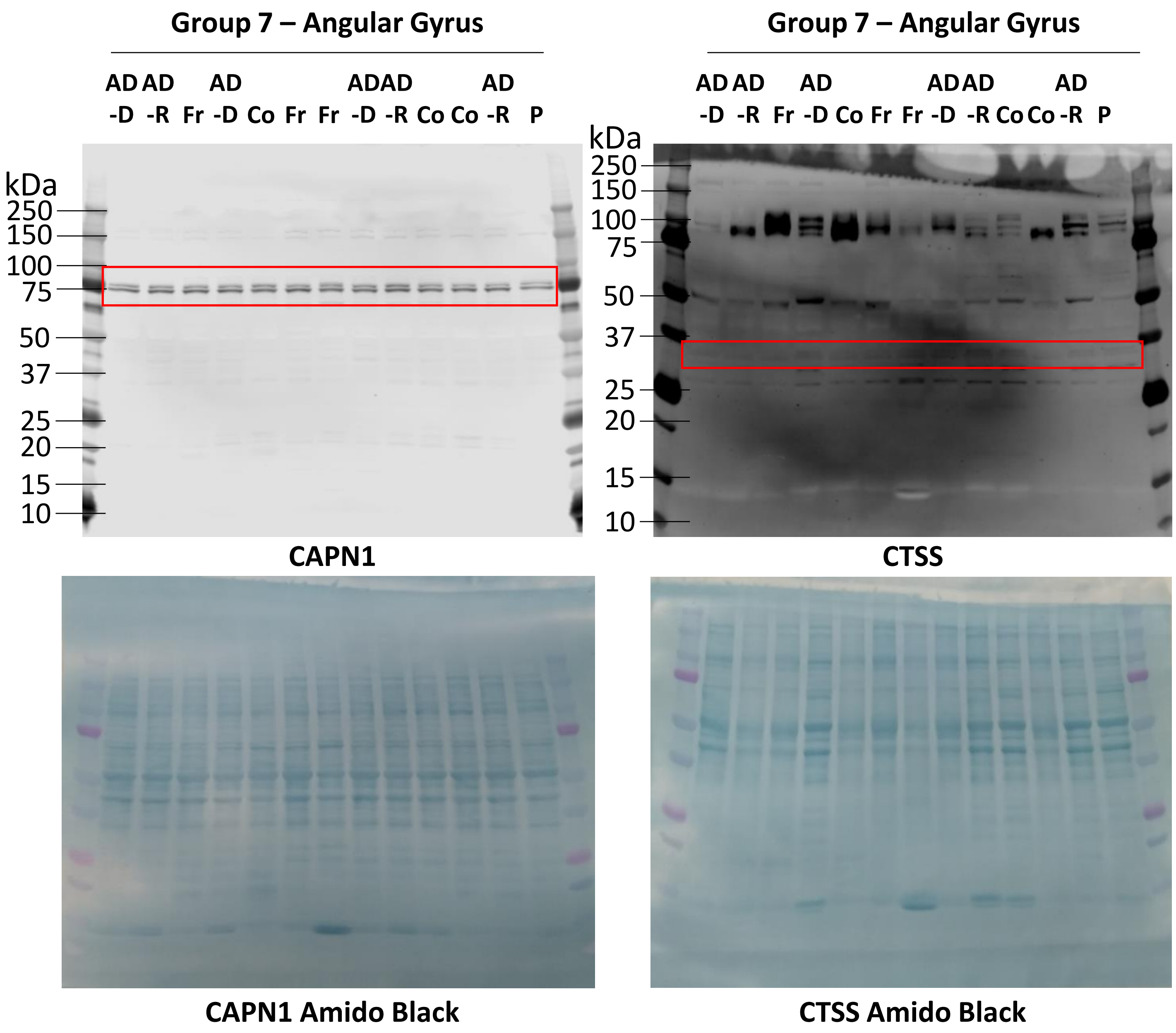

Supplementary Figure 13: All immunoblots and amido black stains from brain protease analysis. Angular Gyrus samples were harvested from the four diagnostic groups, Control (Co, n = 21), AD-DEM (AD-D, n = 20), AD-Resilient (AD-R, n = 21), Frail (Fr, n = 22) and the pooled sample (P) were separated by SDS-PAGE (20 µg total protein/lane). Samples were blinded and split into seven groups for immunoblot analysis. Nitrocellulose membranes were immunoblotted with CAPN1 and CTSS specific antibodies then were stained with amido black as a loading control, all immunoblots and their respective amido black stains are shown.
