# Supplementary material for: Cerebrospinal Fluid and Brain Proteoforms of the Granin Neuropeptide Family in Alzheimer’s Disease
Source: J Am Soc Mass Spectrom. 2023 Mar 13;34(4):649–67. doi: 10.1021/jasms.2c00341 (PMC10080684; doi:10.1021/jasms.2c00341)
Supplement: Supplementary file 1 — js2c00341_si_001.pdf [file js2c00341_si_001.pdf]

Additional information – Supplementary figure legends

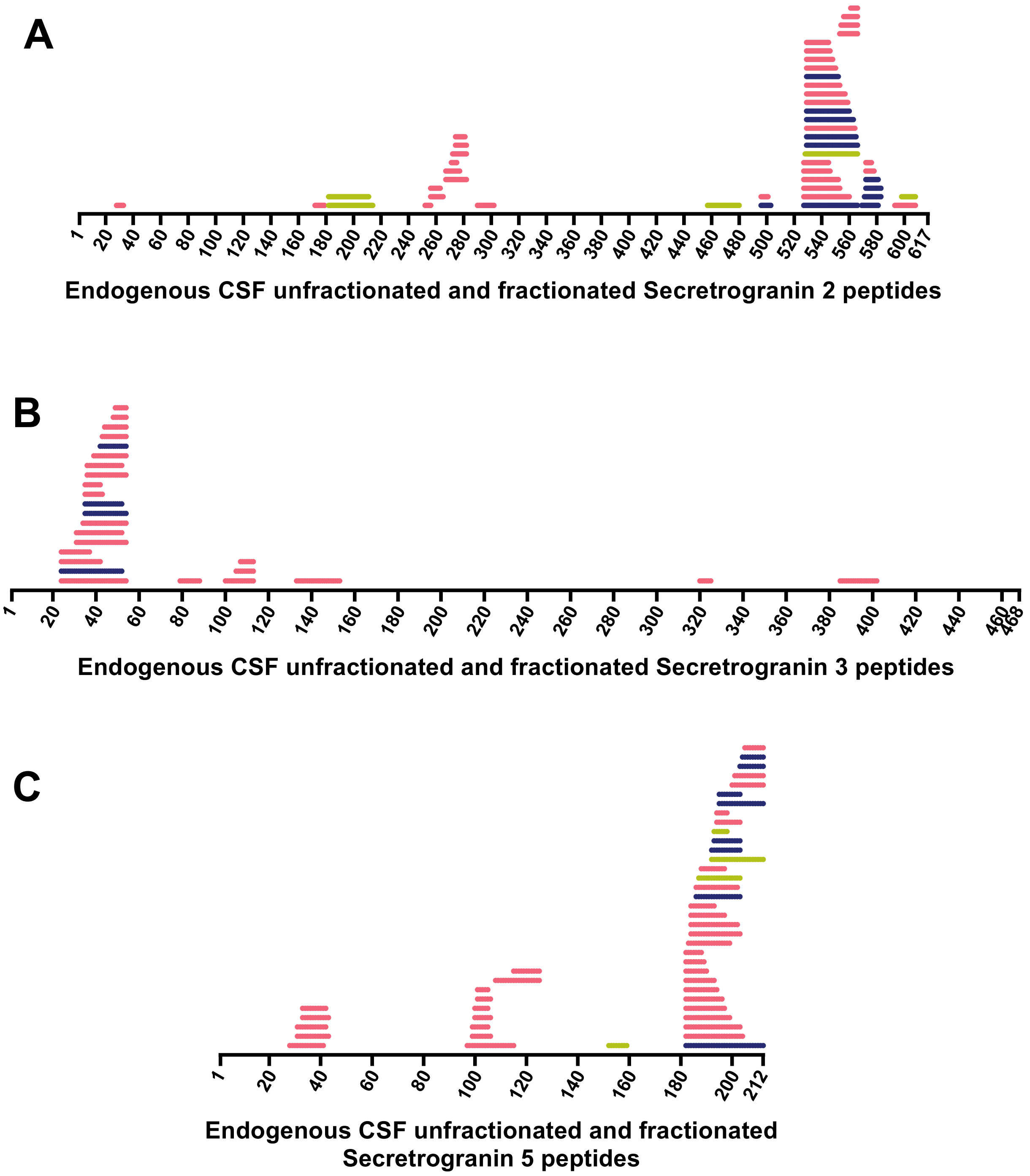

Supplementary Figure 1: Comparison of fractionated and unfractionated CSF analysis of secretogranin proteoforms. (A) Secretogranin 2, **(B)** Secretogranin 3, **(C)** Secretogranin 5. Color key is the same as Figure 1.

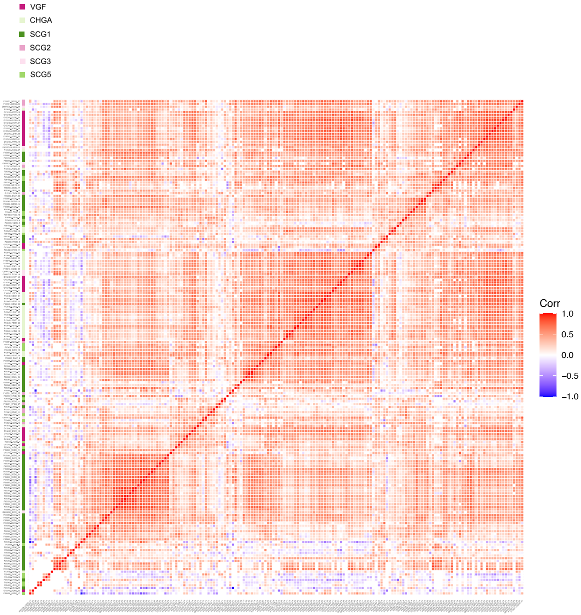

Supplementary Figure 2: Correlation plot of CSF neuropeptide proteoforms. CSF neuropeptides quantified in the 165 CSF sample set show a generally high level of positive correlation both within parent proteins and between different parent proteins. Colored side bar shows the parent protein of the peptide in each row.

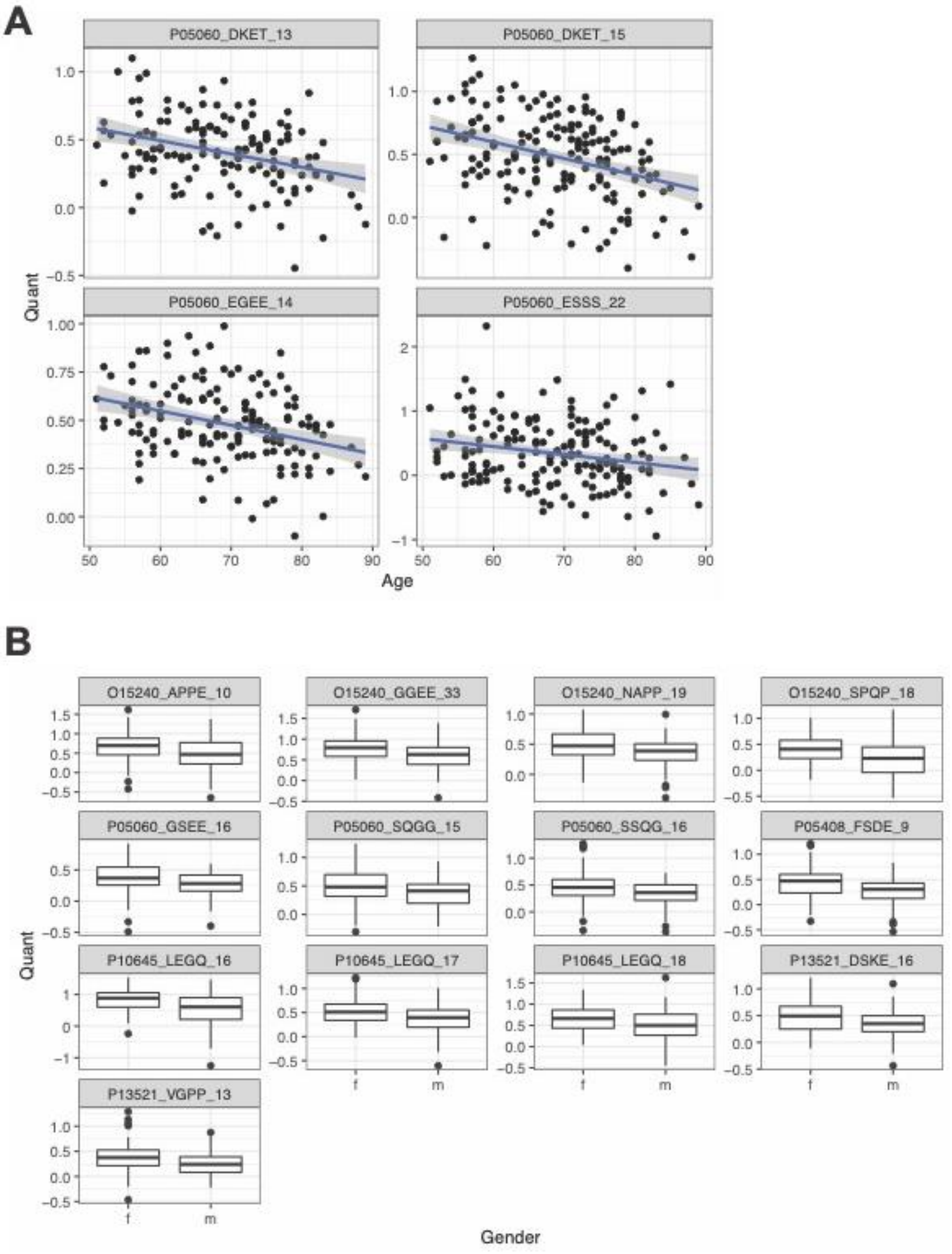

Supplementary Figure 3: Correlation of CSF neuropeptide proteoforms significantly associated with age and sex. (A) Age. **(B)** Sex.

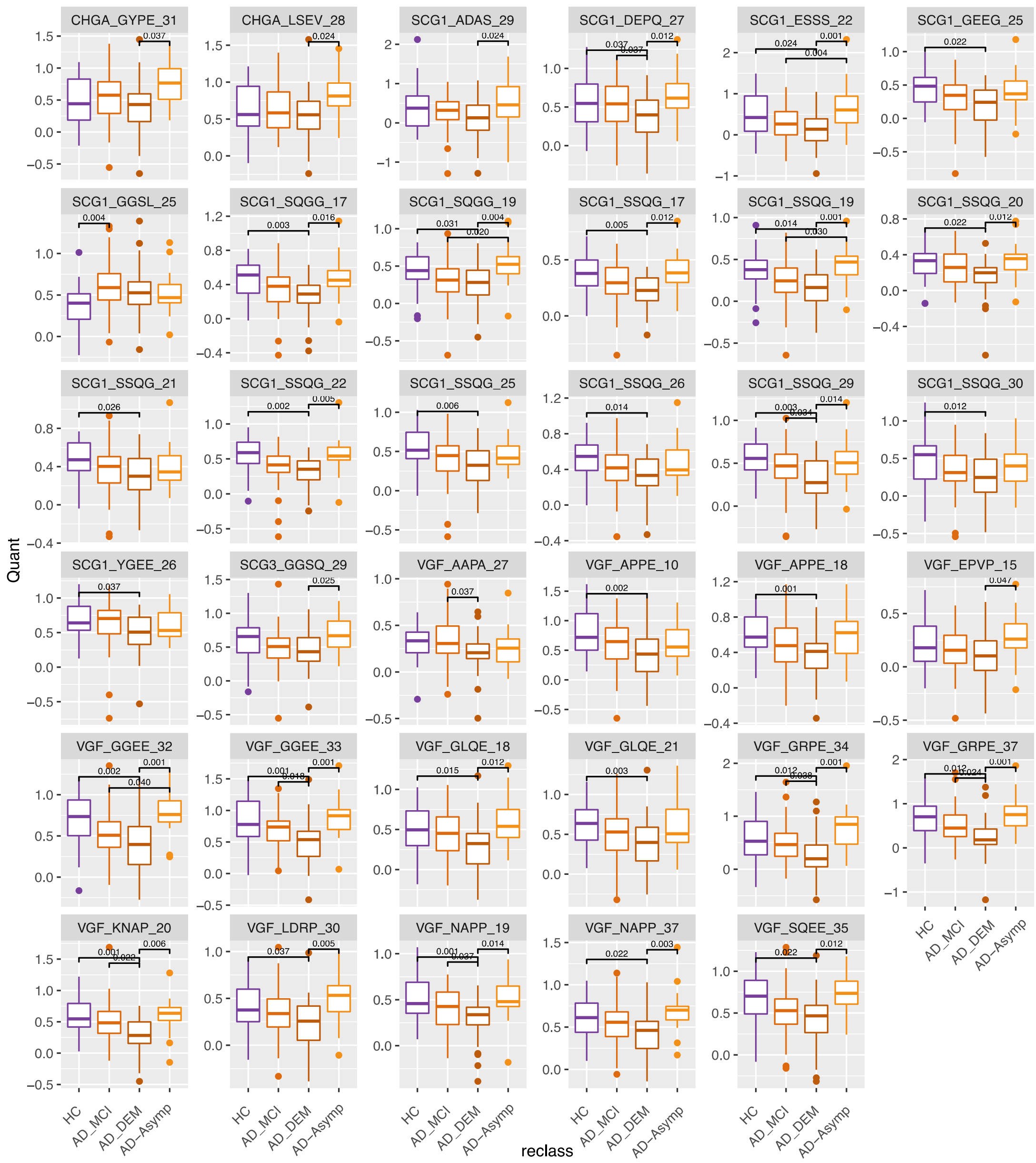

Supplementary Figure 4: All CSF neuropeptide proteoforms that are significantly associated with at least one between group contrast. Benjamini-Hochberg adjusted p values below 0.05 are indicated above the significance bar on each plot.

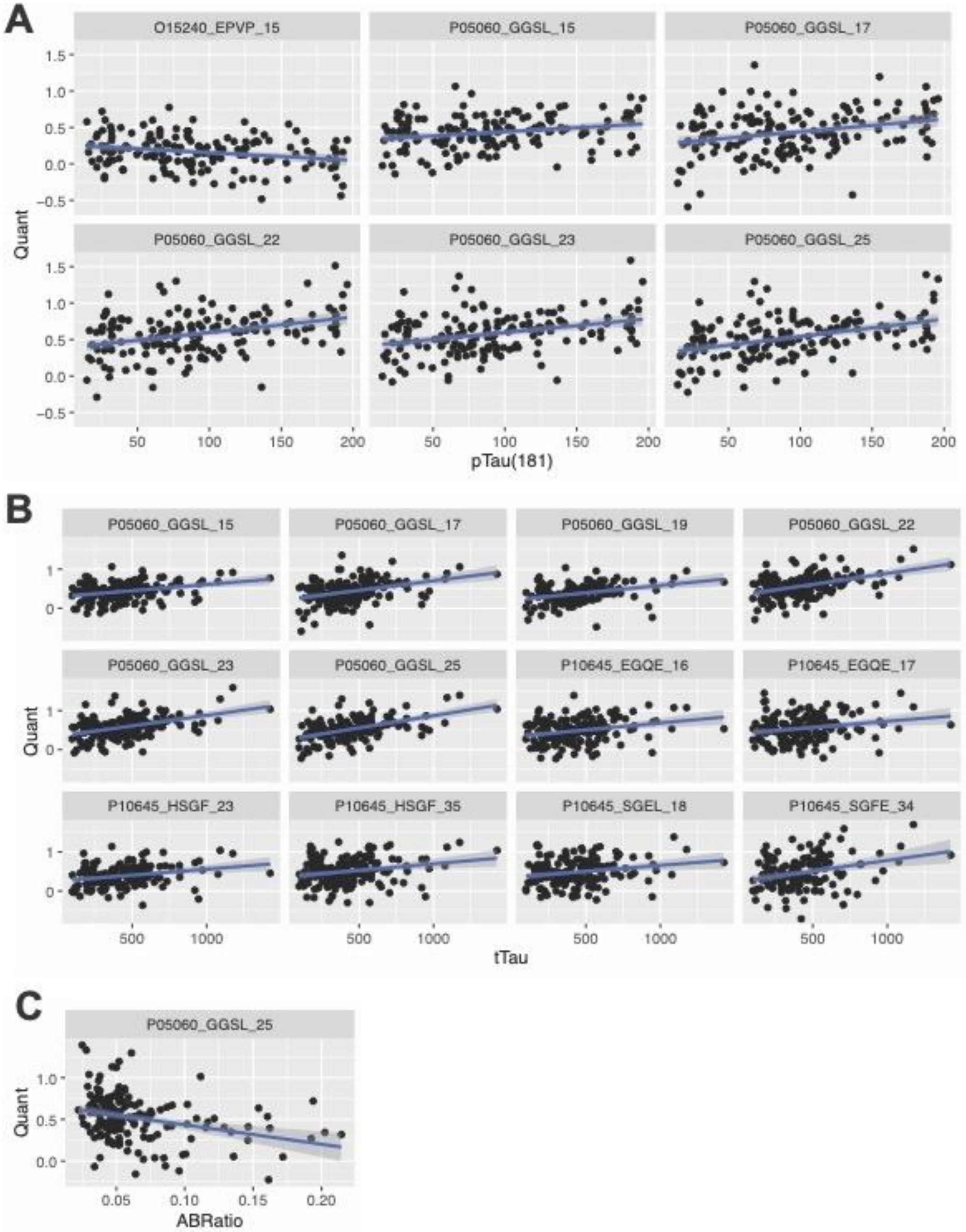

Supplementary Figure 5: Correlation of CSF neuropeptide proteoforms with ATN values.
(A) pTau (181), **(B)** tTau, **(C)** ABRatio. CSF ATN values were measured by EuroImmun ELISAs.

A

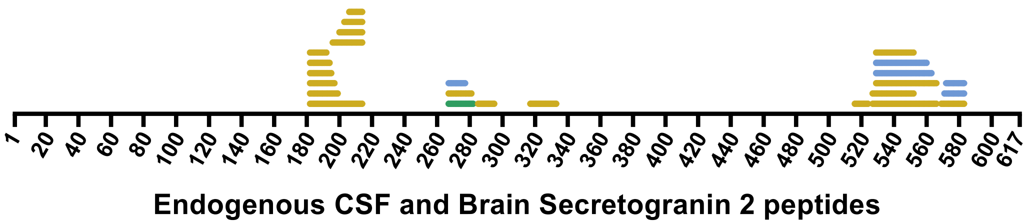

B

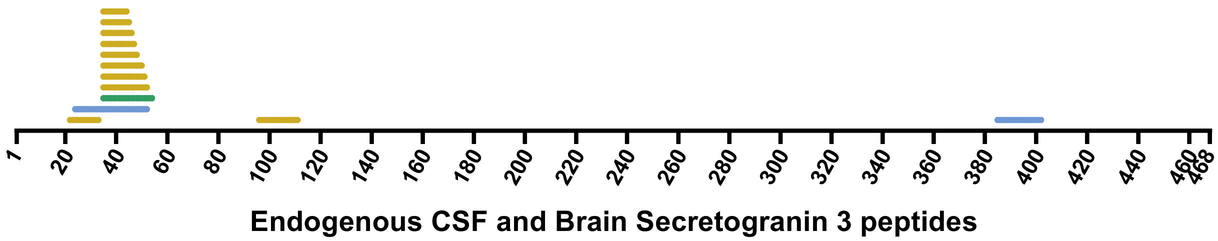

C

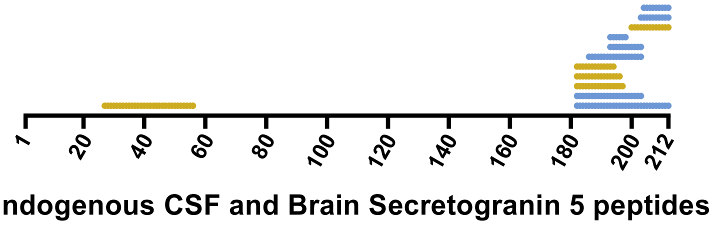

Supplementary Figure 6: Comparison of brain and CSF neuropeptide proteoforms for secretogranins. (A) Secretogranin 2, **(B)** Secretogranin 3, **(C)** Secretogranin 5. Color key is identical to Main Figure 4 maps.

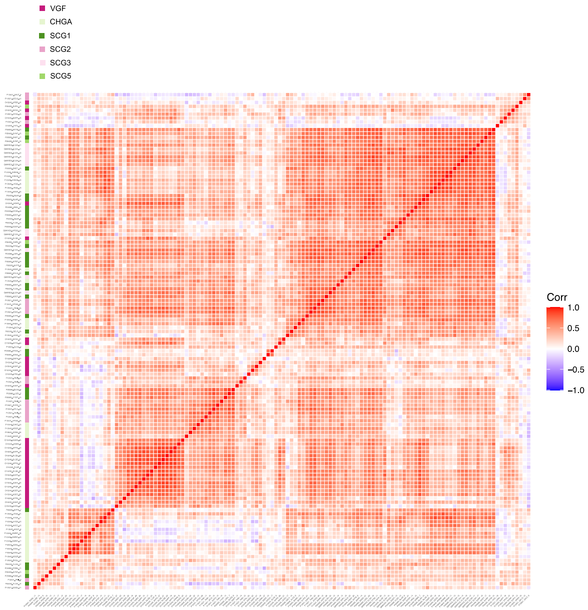

Supplementary Figure 7: Correlation plot of brain neuropeptide proteoforms. Brain neuropeptide proteoforms quantified in the 102-brain sample set show a moderately high level of positive correlation both within parent proteins and between different parent proteins. Colored side bar shows the parent protein of the peptide in each row.

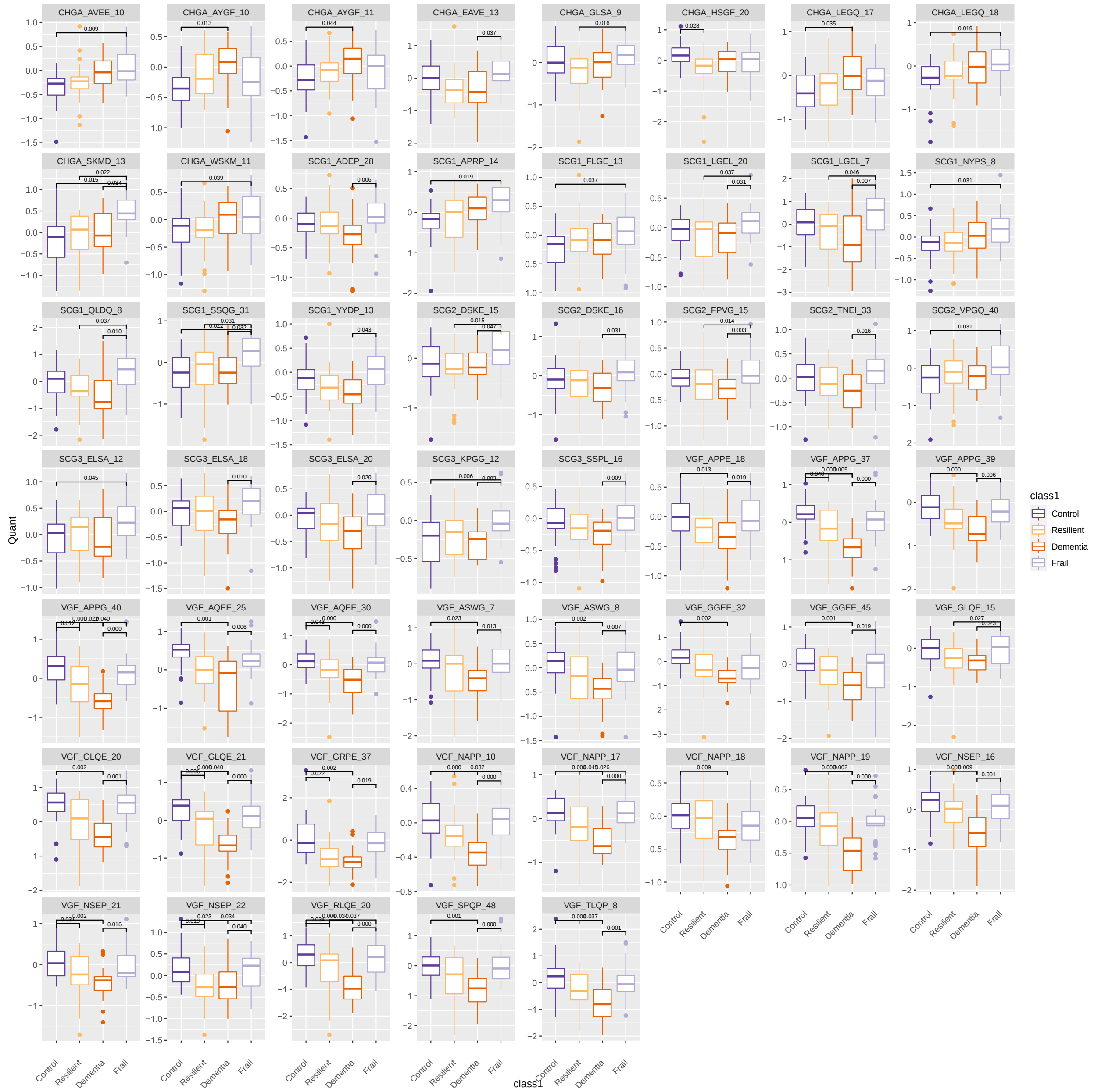

Supplementary Figure 8: All brain neuropeptide proteoforms that are significantly associated with at least one between group contrast. Benjamini-Hochberg adjusted p values below 0.05 are indicated above the significance bar on each plot.

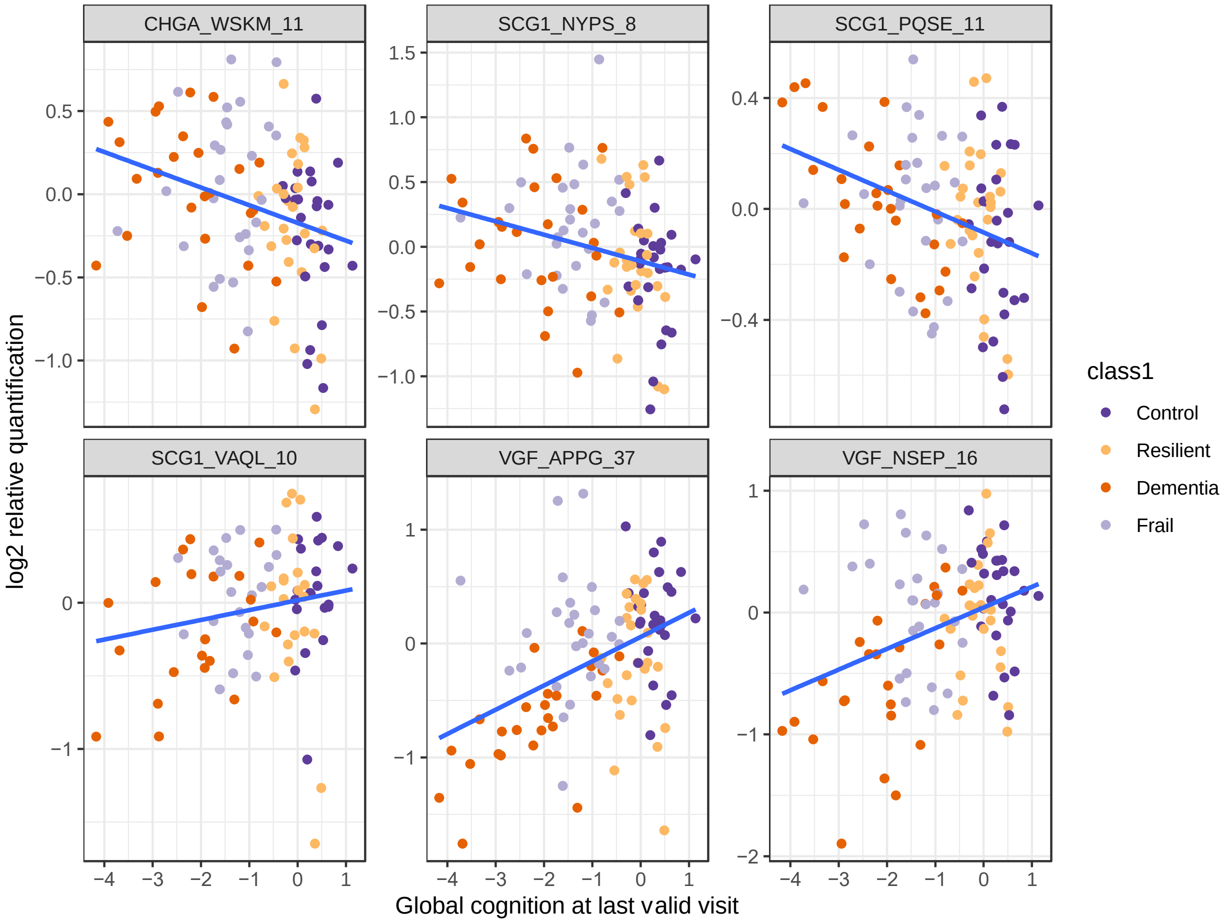

Supplementary Figure 9: Correlation of brain neuropeptide proteoforms with cognition. These six peptides, when included in a predictive model of global cognition, significantly improve model performance over the base model.

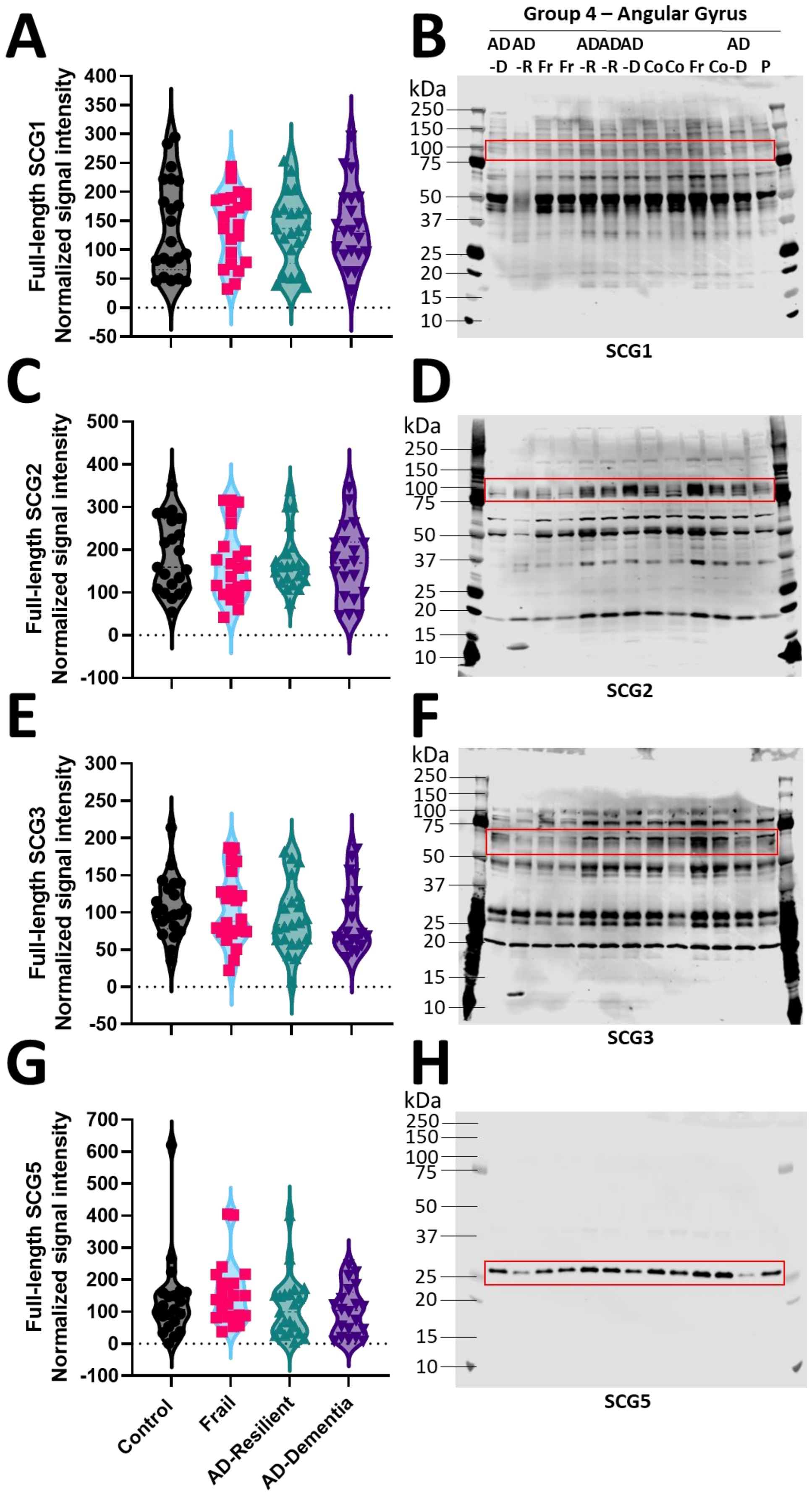

A
B
Group 4 – Angular Gyrus
AD -D
AD -R
Fr
Fr
AD -R
AD -R
AD -D
Co
Co
Fr
Co
AD -D
P
kDa
250
150
100
75
50
37
25
20
15
10
SCG1
Full-length SCG1
Normalized signal intensity
400
350
300
250
200
150
100
50
0
-50
C
D
Full-length SCG2
Normalized signal intensity
500
400
300
200
100
0
-100
SCG2
E
F
Full-length SCG3
Normalized signal intensity
300
250
200
150
100
50
0
-50
SCG3
G
H
Full-length SCG5
Normalized signal intensity
700
600
500
400
300
200
100
0
-100
SCG5
Control
Frail
AD-Resilient
AD-Dementia

Supplementary Figure 10: Quantification of tissue full-length secretogranin levels. (A, C, E, G) Semi-quantitative densitometry of fluorescence was performed following immunoblotting using LI-COR Image Studio Lite. Data are expressed as the ratio of full-length SCG1 **(A)**, SCG2 **(C)**, SCG3 **(E)** and SCG5 **(G)** to the pooled sample relative to the amido stain. Data are visualized by violin plot with all points shown, n=84. Data were analysed using the Kruskal-Wallis test with the Dunn's test for multiple comparisons. **(B, D, F, H)** Angular Gyrus samples were harvested from the four diagnostic groups, Control (Co, n = 21), AD-DEM (AD-D, n = 20), AD-Resilient (AD-R, n = 21), Frail (Fr, n = 22) and the pooled sample (P) were separated by SDS-PAGE (20 µg total protein/lane). Nitrocellulose membranes were immunoblotted with SCG1 **(B)**, SCG2 **(D)**, SCG3 **(F)** and SCG5 **(H)** specific antibodies then were stained with amido black as a loading control, representative images of the immunoblots are shown. The red band indicates the full-length secretogranin analysed **(B, D, F, H)**. Significant value threshold was set with a p value of less than 0.05.

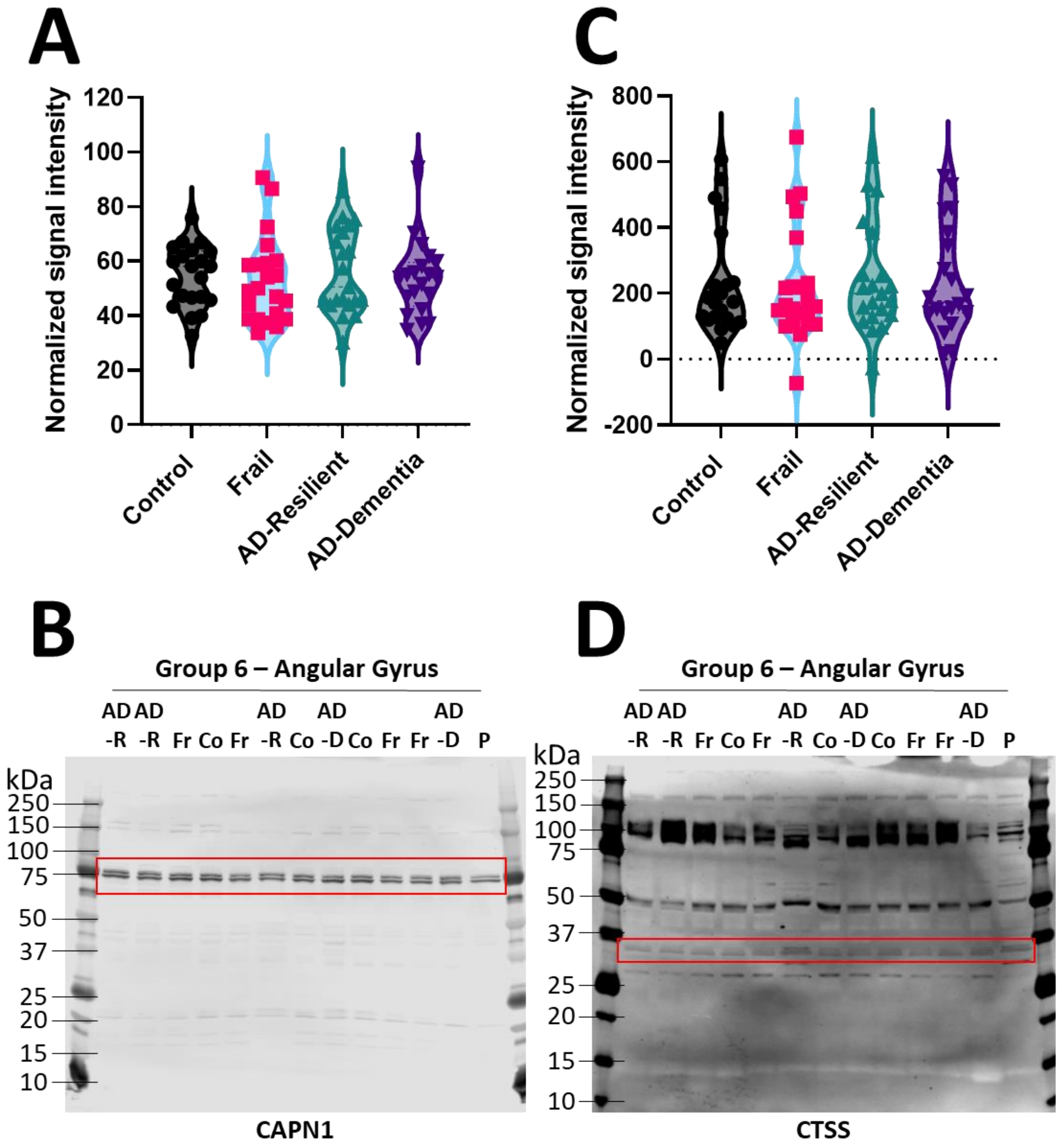

Supplementary Figure 12: Quantification of tissue full-length protease levels. (A, C) Semi-quantitative densitometry of fluorescence was performed following immunoblotting using LI-COR Image Studio Lite. Data are expressed as the ratio of full-length CAPN1 **(A)** and CTSS **(C)** to the pooled sample relative to the amido stain. Data are visualized by violin plot with all points shown, n=84. Data were analysed using the Kruskal-Wallis test with the Dunn's test for multiple comparisons. **(B, D)** Angular Gyrus samples were harvested from the four diagnostic groups, Control (Co, n = 21), AD-DEM (AD-D, n = 20), AD-Resilient (AD-R, n = 21), Frail (Fr, n = 22) and the pooled sample (P) were separated by SDS-PAGE (20 µg total protein/lane). Nitrocellulose membranes were immunoblotted with CAPN1 **(B)** and CTSS **(D)** specific

antibodies then were stained with amido black as a loading control, representative images of the immunoblots are shown. The red band indicates the full-length protease analysed **(B, D)**. Significant value threshold was set with a p value of less than 0.05.
